# Supplementary material for: Quasi-solid-state Zn-air batteries with an atomically dispersed cobalt electrocatalyst and organohydrogel electrolyte
Source: Nat Commun. 2022 Jun 27;13:3689. doi: 10.1038/s41467-022-31383-4 (PMC9237111; doi:10.1038/s41467-022-31383-4)
Supplement: Supplementary file 1 — Unmarked Supplementary Information [file 41467_2022_31383_MOESM1_ESM.docx]

## Supplementary Information for

**Quasi-solid-state Zn-air batteries with an atomically dispersed cobalt electrocatalyst and organohydrogel electrolyte**

Wang et al.

In generally, battery polarization at low temperature includes the following three aspects: activation polarization, ohmic polarization, and concentration polarization. As known, the battery voltage is mainly affected by both polarization and internal resistance. Both of polarization and internal resistance are severely restricted by working temperature. In the low-potential window that just over the equilibrium potential, the electrochemical reaction is mainly in a state where the molecules are activated to allow the reaction to proceed. The overpotential is mainly used to overcome the activation energy of the reaction, causing the activation polarization. The decrease of electrolyte ionic conductivity, the electron transport in the electrode and interface also decreases at low temperatures, which result in huge increase in the Ohmic polarization. At high applied potential, the ion diffusion rate of electrolyte is slower than the electrochemical reaction rate due to the mass transport limitation, resulting in the concentration polarization.

The output voltage is a critical parameter in electrochemical batteries. Based on the second law of thermodynamics, low temperate undoubtedly lower the charge (ion and electron) transport in electrode and electrolyte, leading to a considerable resistance enhancement.

**From the point of thermodynamics factor:**

$\text{ΔG = -}\text{nEF}$ (1)

Where *ΔG* is the Gibbs free energy, n is the of transferred electron number, *E* is the battery voltage and *F* is the Faraday constant. Nernst equation describes the relationship between battery voltages and working temperature could be established as follow:

$\text{E}\text{ }\text{=}\text{ }\text{E}^{\text{ø}\text{ }}\text{-}\text{ }\frac{\text{RT}}{\text{nF}}\text{In}\frac{\text{[C]}^{\text{c}}\text{[D]}^{\text{d}}}{\text{[A]}^{\text{a}}\text{[B]}^{\text{b}}}$ (2)

In this equation, *E* stands for the actual measured voltage, $\text{E}^{\text{ø}}$ represents the battery voltage at the standard state, *T* is the working temperature and *R*, *n*, and *F* are all constants. In general, the delivered voltage conforms to the Nernst equation. When the battery is discharging by electrochemical reactions, $\frac{\text{[C]}^{\text{c}}\text{[D]}^{\text{d}}}{\text{[A]}^{\text{a}}\text{[B]}^{\text{b}}}$ < 0, $\text{-}\frac{\text{RT}}{\text{nF}}\text{In}\frac{\left[ \text{C} \right]^{\text{c}}\left[ \text{D} \right]^{\text{d}}}{\left[ \text{A} \right]^{\text{a}}\left[ \text{B} \right]^{\text{b}}}\text{ }$> 0 would be obtained. The decrease in *T* leads to a decrease in$\text{-}\frac{\text{RT}}{\text{nF}}\text{In}\frac{\left[ \text{C} \right]^{\text{c}}\left[ \text{D} \right]^{\text{d}}}{\left[ \text{A} \right]^{\text{a}}\left[ \text{B} \right]^{\text{b}}}$, which will cause a decrease in the *E*. Consequently, the decrease in delivered voltage results in the energy efficiency loss of battery.

**From the point of kinetic factor:**

The ionic conductivity is another dominant aspect in electrochemical battery kinetics. The ionic diffusivity (*D_i_*) is commonly used to quantitatively assess the movement of ions. The connection between the Arrhenius parameter and the ionic diffusivity are as follow:

$\text{D}_{\text{i}}\text{= }\text{D}_{\text{0}}\exp\left( \text{-}\frac{\text{Δ}\text{G}^{\text{θ}}}{\text{k}_{\text{B}}\text{T}} \right)\text{= }\frac{\text{ζ}^{\text{2}}}{\text{t}}$ (3)

where *D_0_* is the estimated pre-factor, *ΔG^θ^* is the energy barrier, *k_B_* is the Boltzmann constant, and *ζ* is the diffusion length. The *D_i_* is also related to the diffusion time (*t*).

**Supplementary Figures**
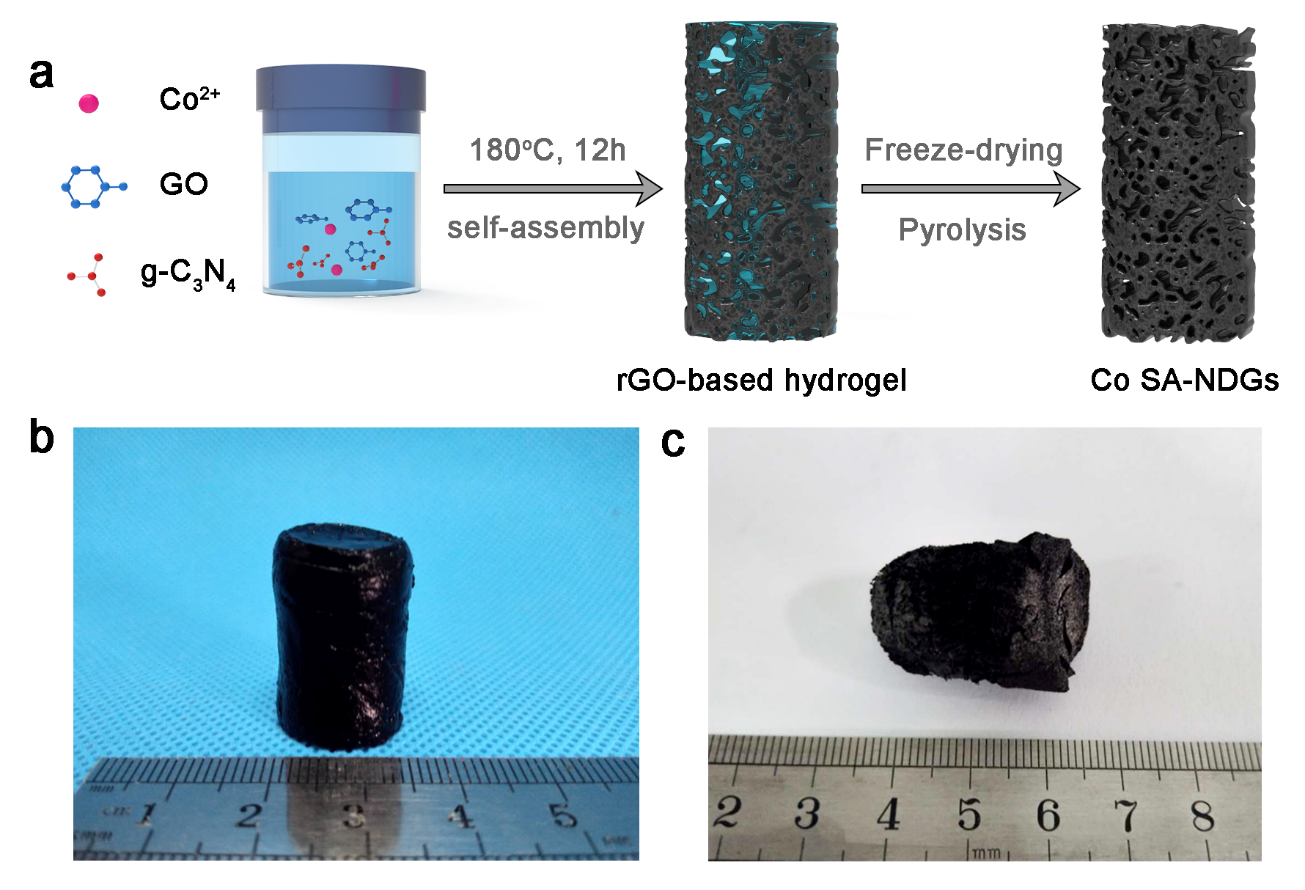


**Supplementary** **Figure 1**. (a) Schematic illustration of the fabrication process for Co SA-NDGs. Optical photographs of (b) rGO-based hydrogel and (c) Co SA-NDGs.


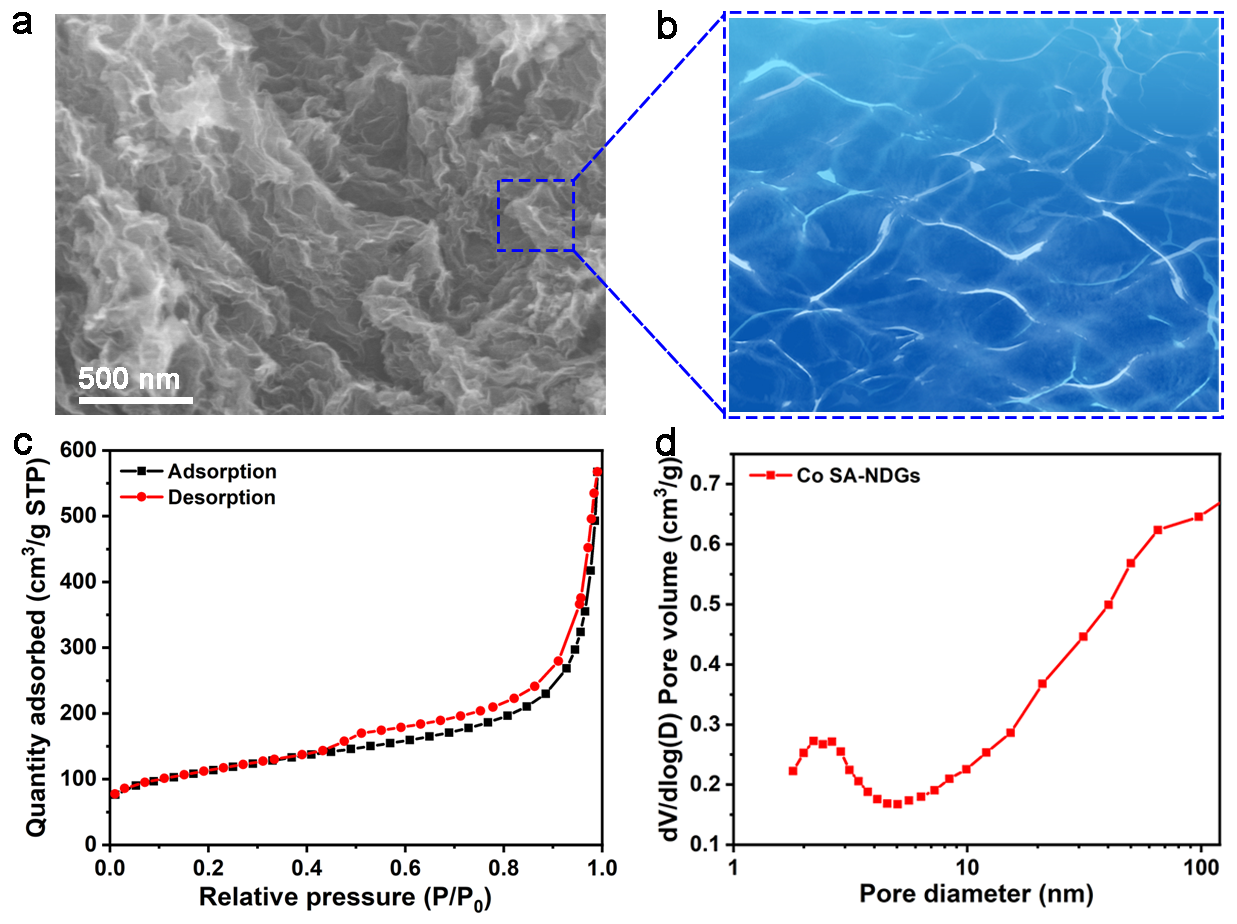


**Supplementary Figure 2**. (a) SEM image of Co SA-NDGs. (b) Fluctuating sea level. (c) N_2_ adsorption/desorption isotherms and (d) corresponding pore size distribution of Co SA-NDGs.


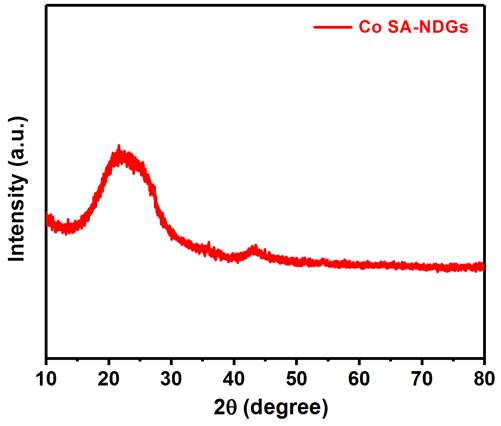


**Supplementary Figure 3**. XRD pattern of Co SA-NDGs.


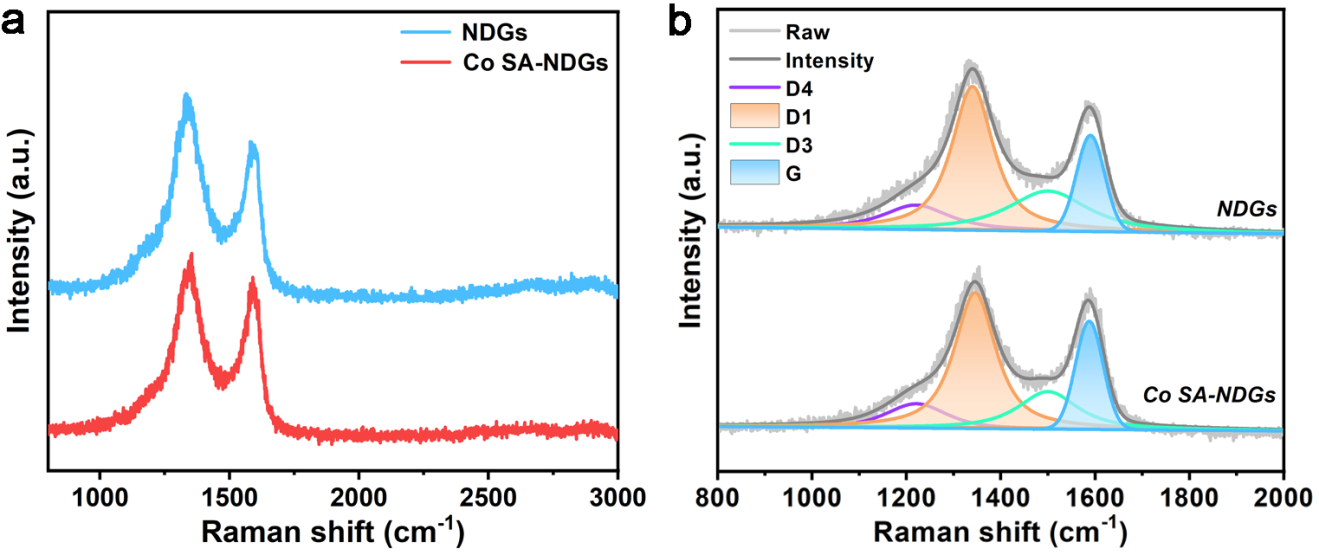


**Supplementary Figure 4**. (a) Raman spectrum and (b) Curve fitting results of NDGs and Co SA-NDGs.

Two broad peaks (about 1350 and 1580 cm^-1^) were observed, corresponding to the D-band and G-band, respectively. To obtain valuable information of the structural defects, the Raman spectra have been fitted with four bands (D1, D3, D4, and G)^1^. Specifically, the G band corresponds to the defect-free sp^2^ carbon. The D1 band is associated with the small crystallite sizes, grains, or edge plane defects of graphite domains. The D3 band is assigned to amorphous carbon, while the presence of D4 band has been tentatively related to the existence of polyene-like structures or ionic impurities. The intensity ratio of D1 and G band of Co SA-NDGs is high up to 3.13, suggesting the abundant structural defects (e.g., edge and in-plane atomic vacancy, etc).


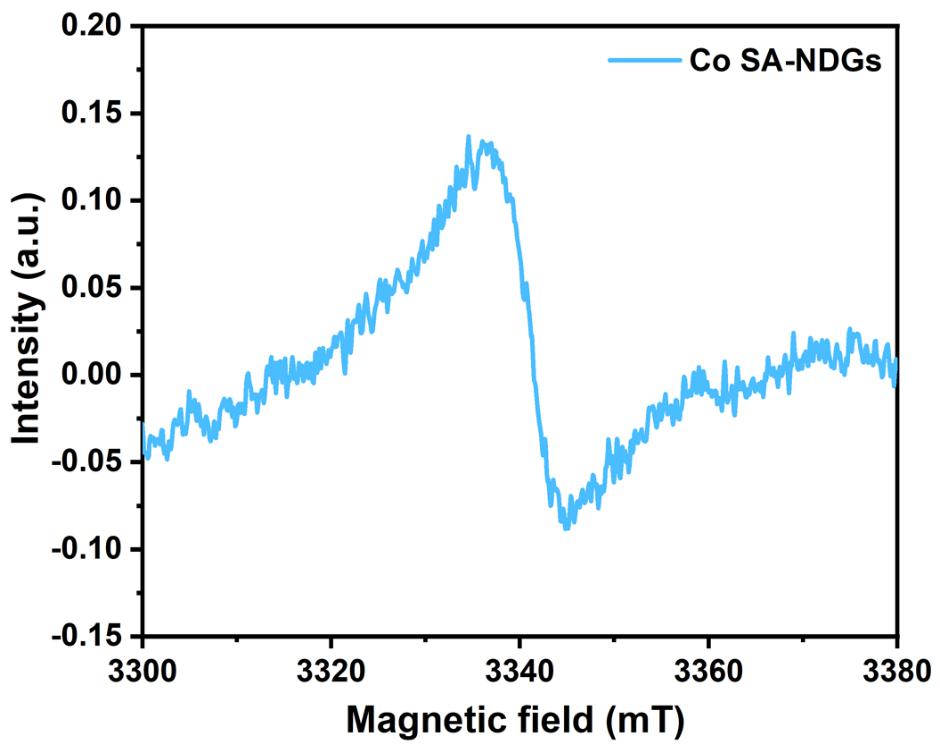


**Supplementary Figure 5**. EPR spectrum of Co SA-NDGs.


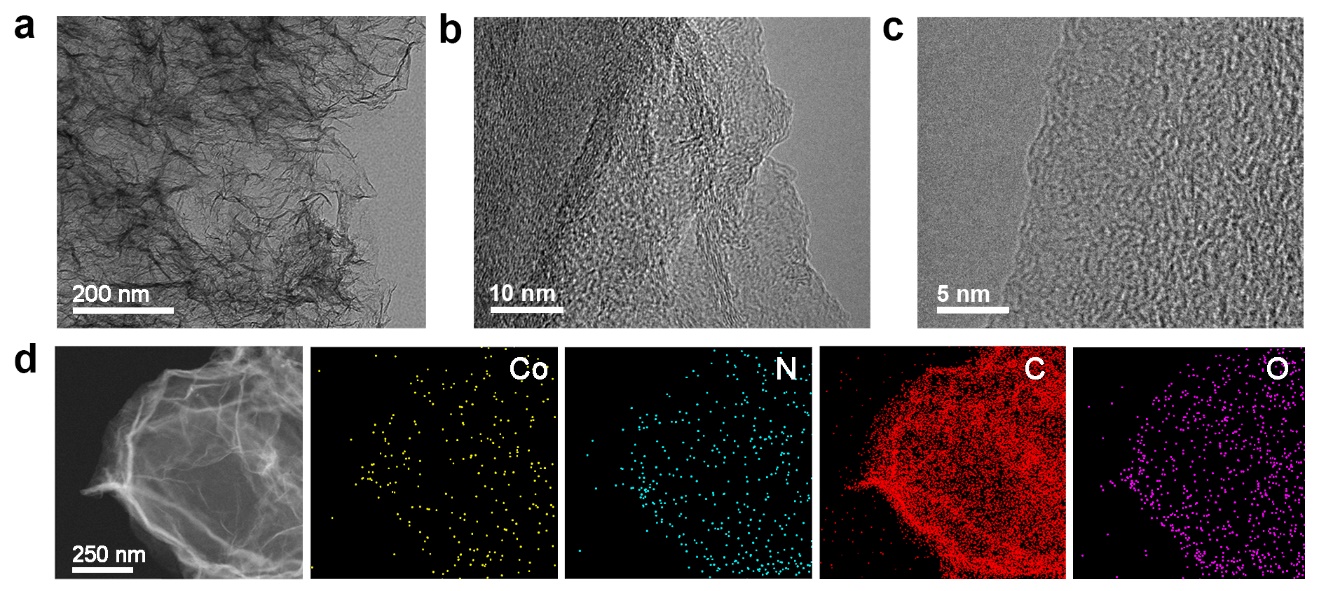


**Supplementary Figure 6**. (a-c) TEM and HRTEM images of Co SA-NDGs. (d) STEM image and element mapping images of Co SA-NDGs.


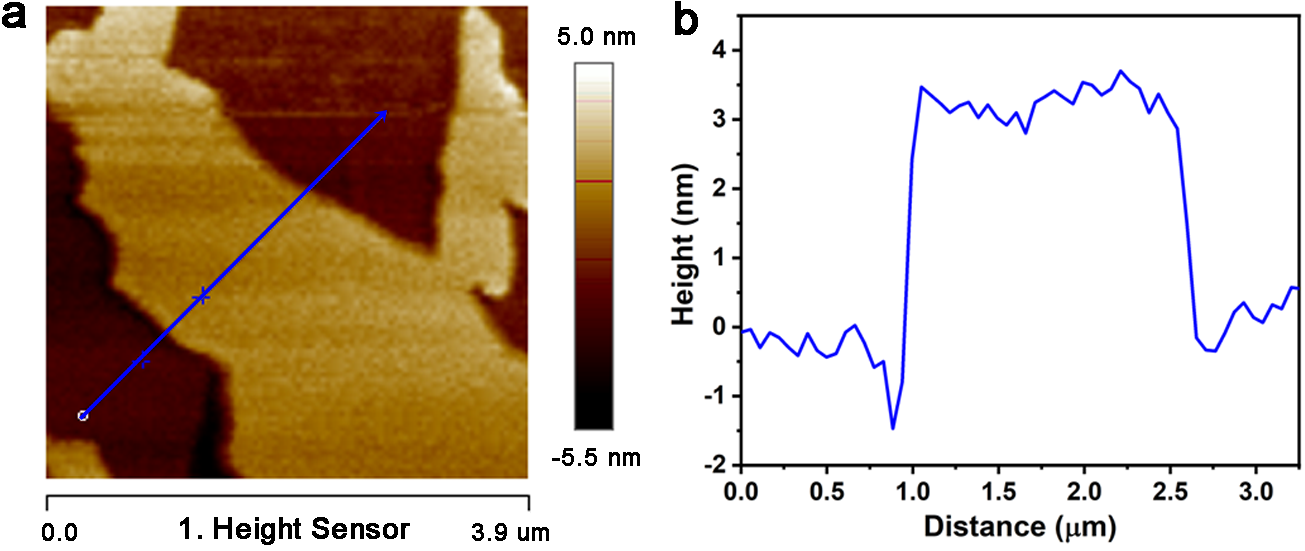


**Supplementary Figure 7**. (a) AFM image of Co SA-NDGs. (b) The height profiles corresponding to the AFM image.


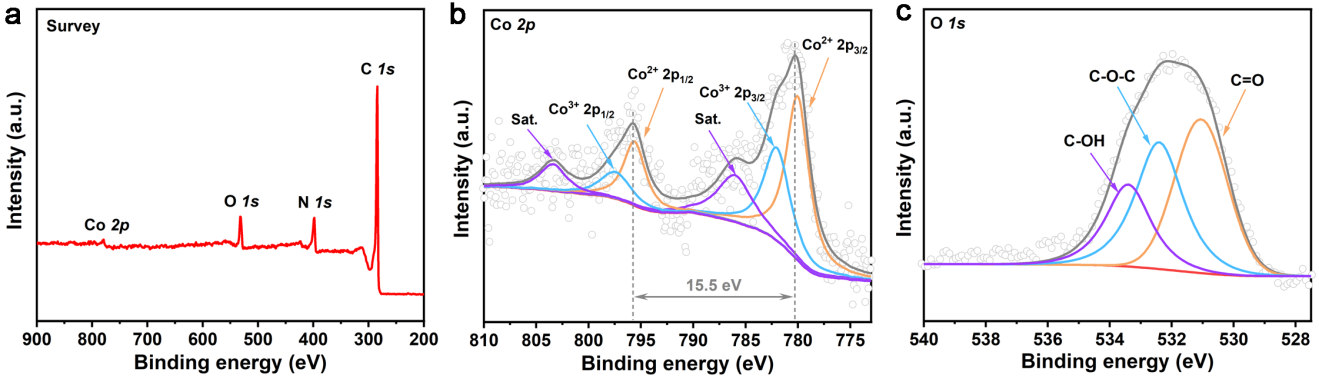


**Supplementary Figure 8**. (a) XPS survey spectrum, (b) Co *2p* spectrum and (c) O *1s* spectrum of Co SA-NDGs.

**
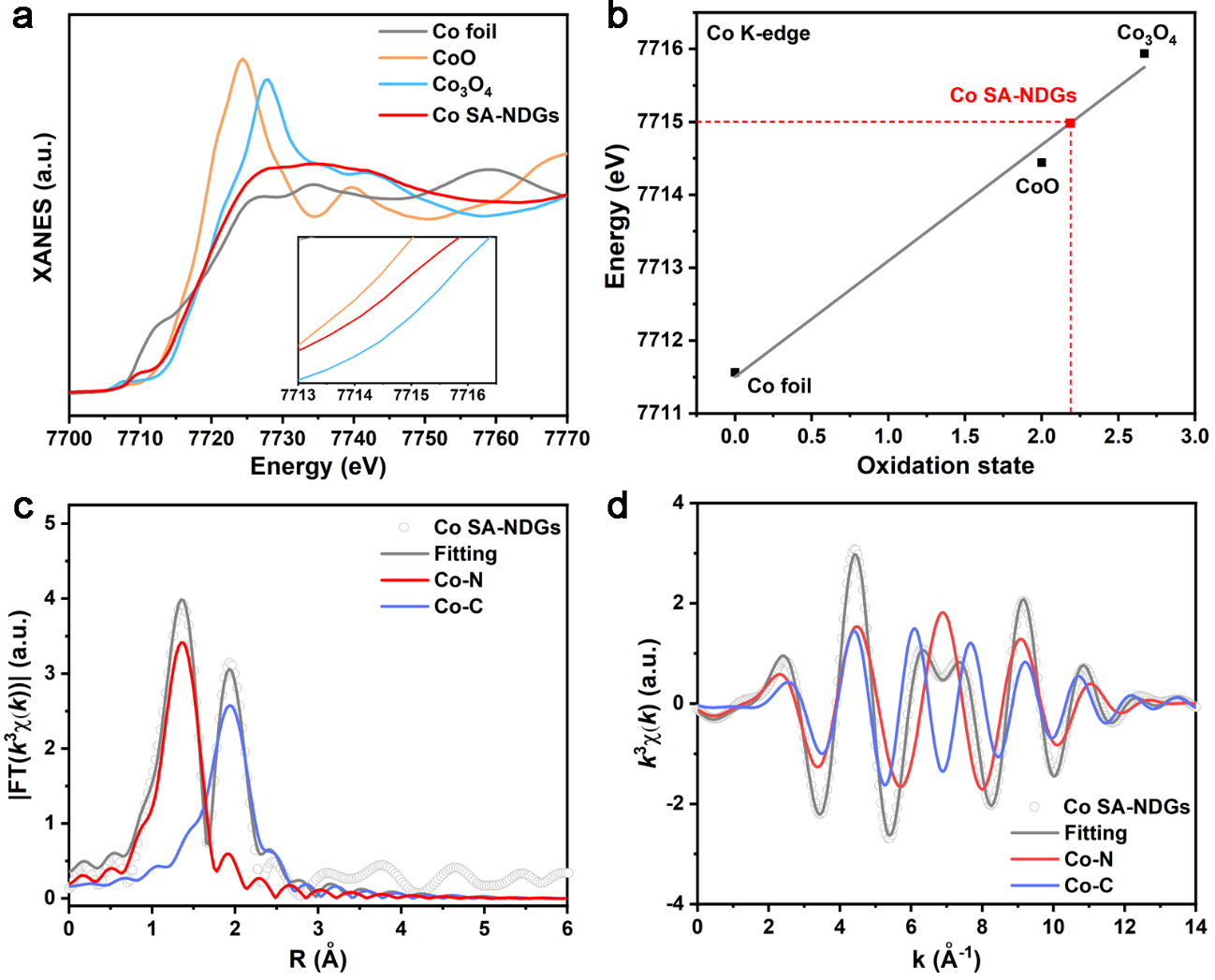
**

**Supplementary Figure 9**. Normalized Co K-edge XANES spectra of Co foil, CoO, Co_3_O_4_, and Co SA-NDGs. (b) The fitted oxidation states of Co. (c) FT of the k^3^-weighted EXAFS spectrum and fitting in R space at the Co K-edge for Co SA-NDGs. (d) EXAFS fitting curves of Co SA-NDGs at k space.


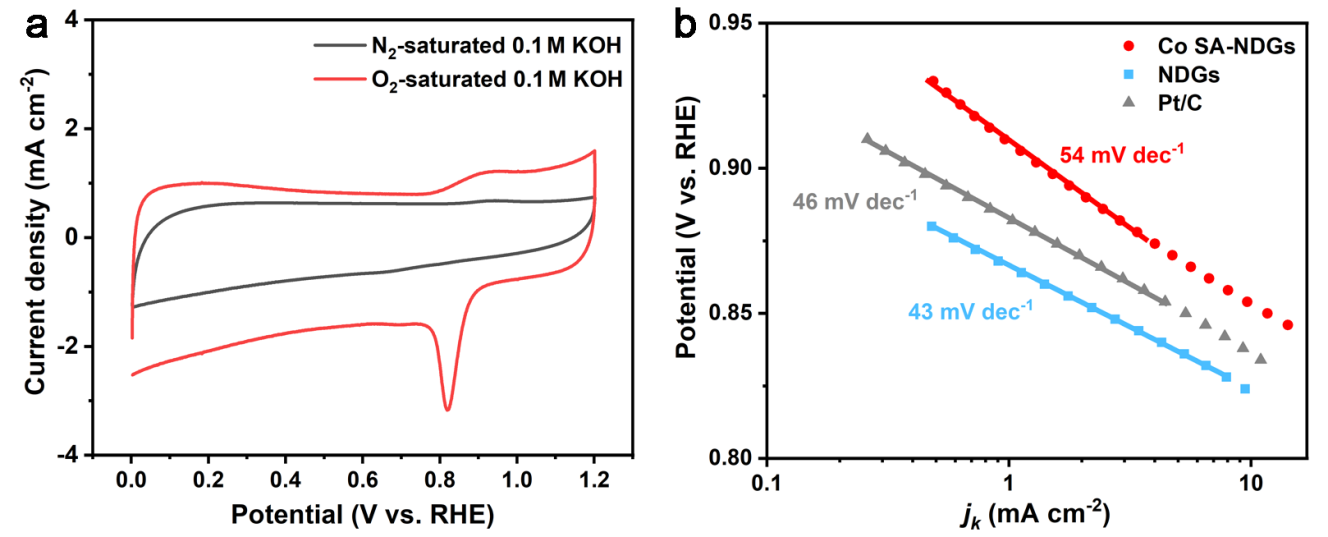


**Supplementary Figure 10**. (a) CV curves of Co SA-NDGs in N_2_/O_2_-saturated 0.1 M KOH. (b) Tafel slopes for NDGs, Co SA-NDGs and Pt/C for ORR.


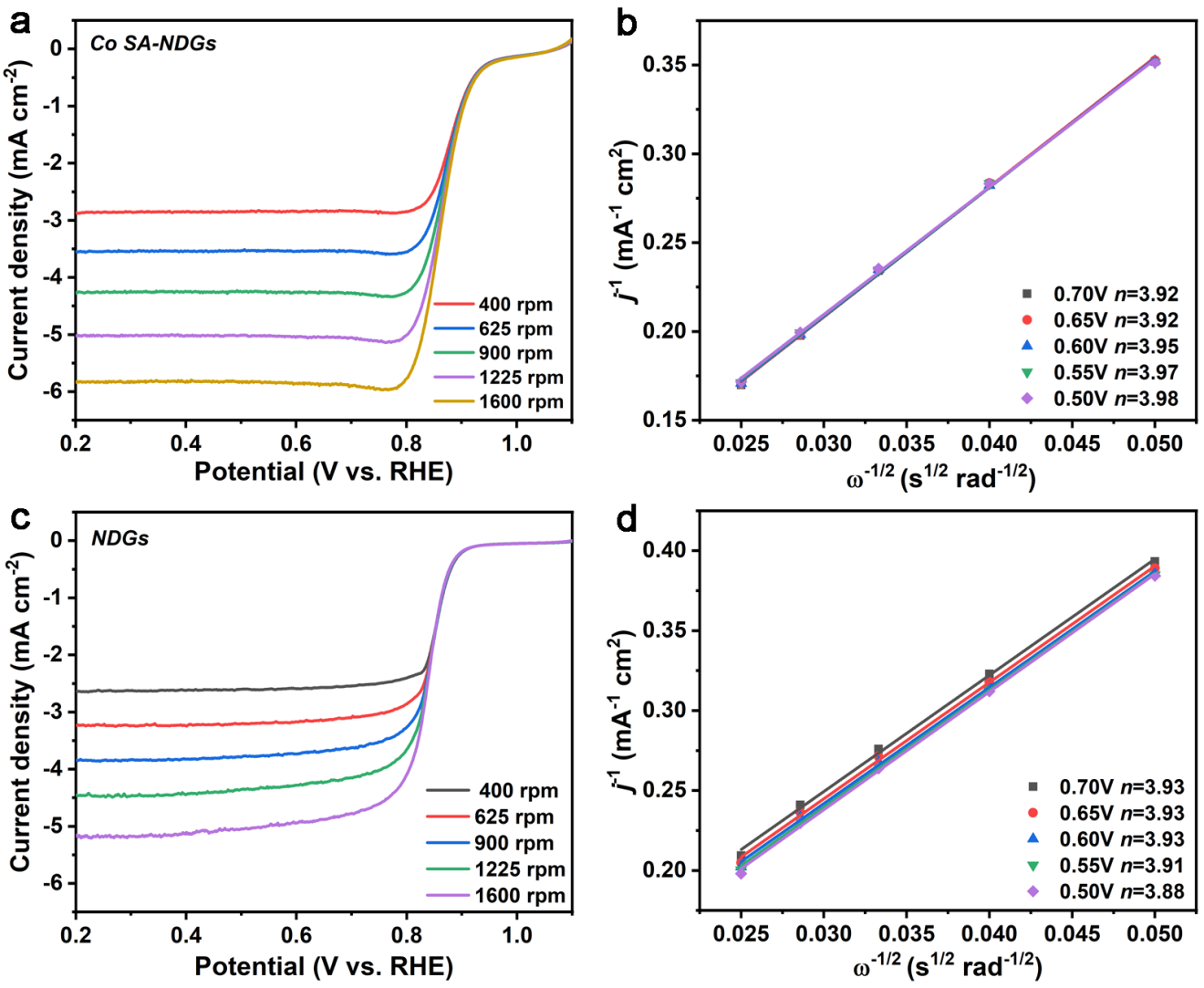


**Supplementary Figure 11**. LSV curves recorded at various rotation rates and K-L plots at different potentials for (a, b) Co SA-NDGs and (c, d) NDGs.


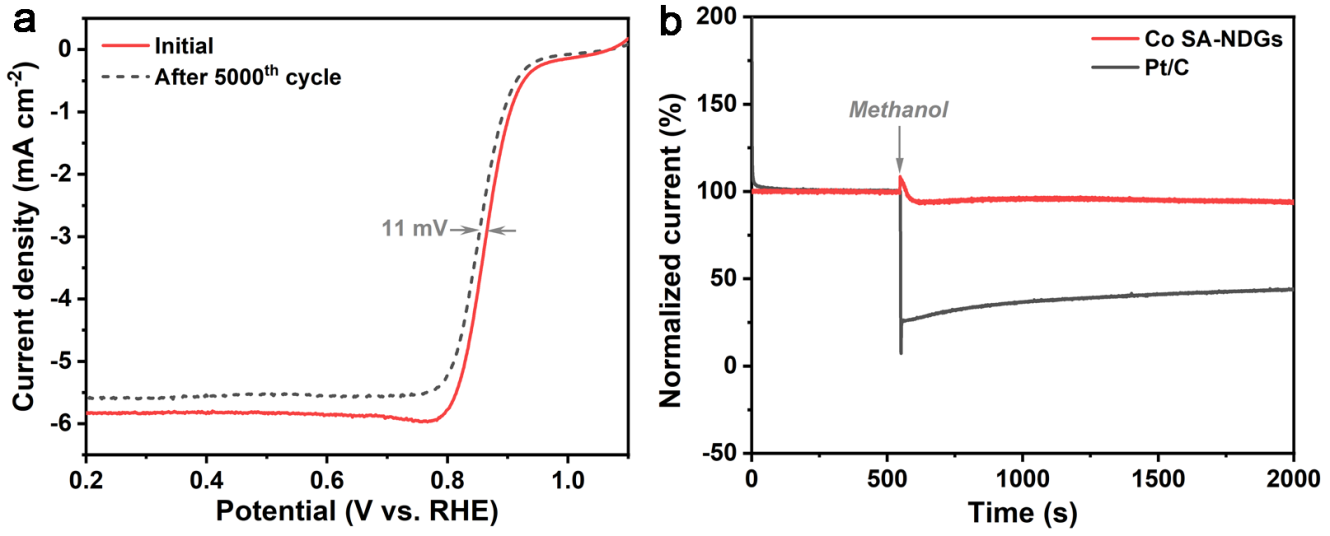


**Supplementary Figure 12**. (a) ORR polarization curves of Co SA-NDGs before and after accelerated degradation measurement. (b) I-t curves of Co SA-NDGs and Pt/C in O_2_-saturated 0.1 M KOH without and with methanol.


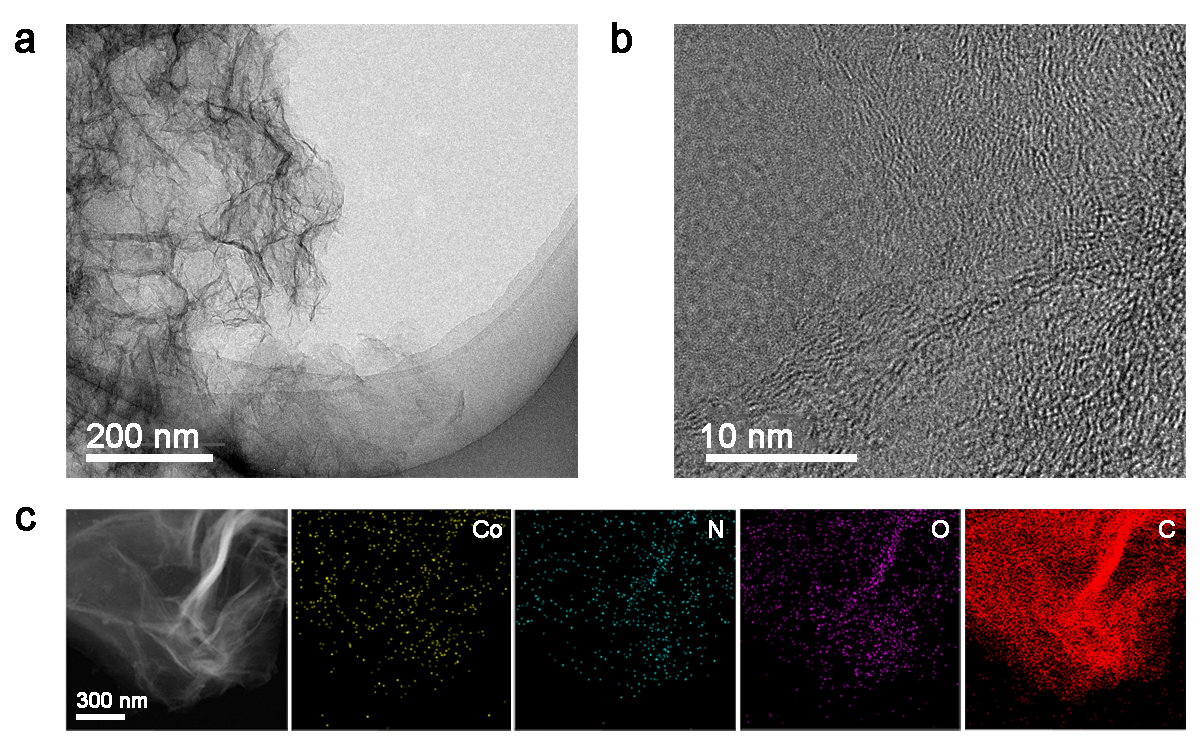


**Supplementary Figure 13**. (a) TEM image and (b) HRTEM image of Co SA-NDGs after ORR stability measurement. (c) STEM image and element mapping images of Co SA-NDGs after ORR stability measurement.


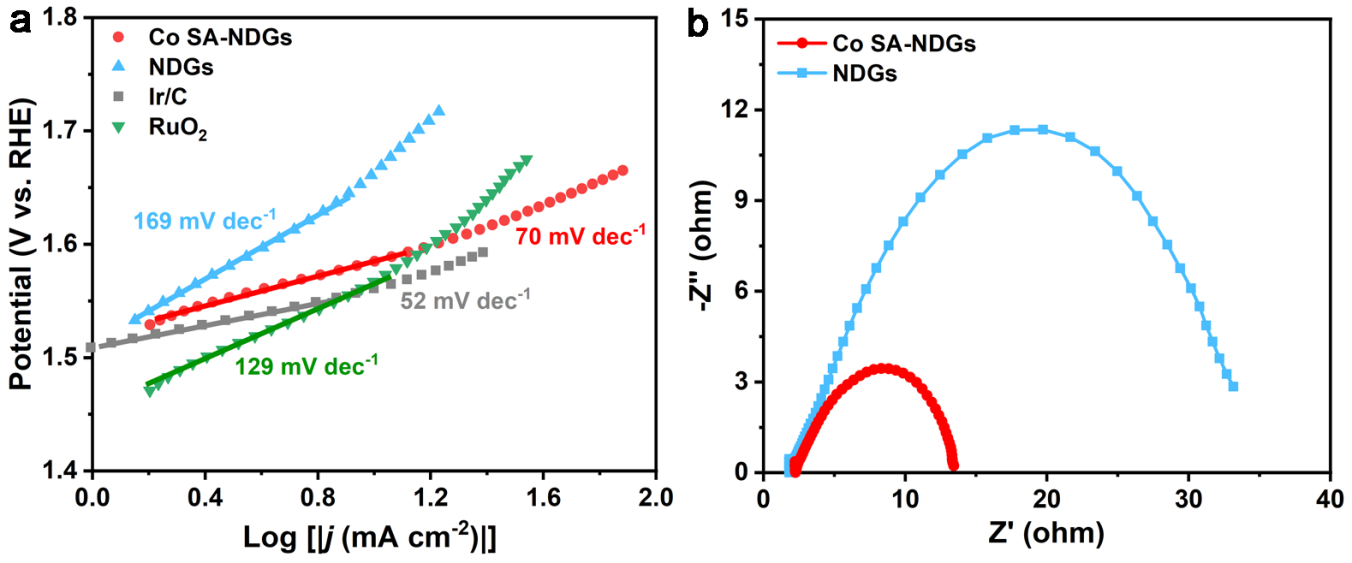


**Supplementary Figure 14**. (a) Tafel slopes of Co SA-NDGs, NDGs, Ir/C and RuO_2_ for OER. (b) EIS results of Co SA-NDGs and NDGs.


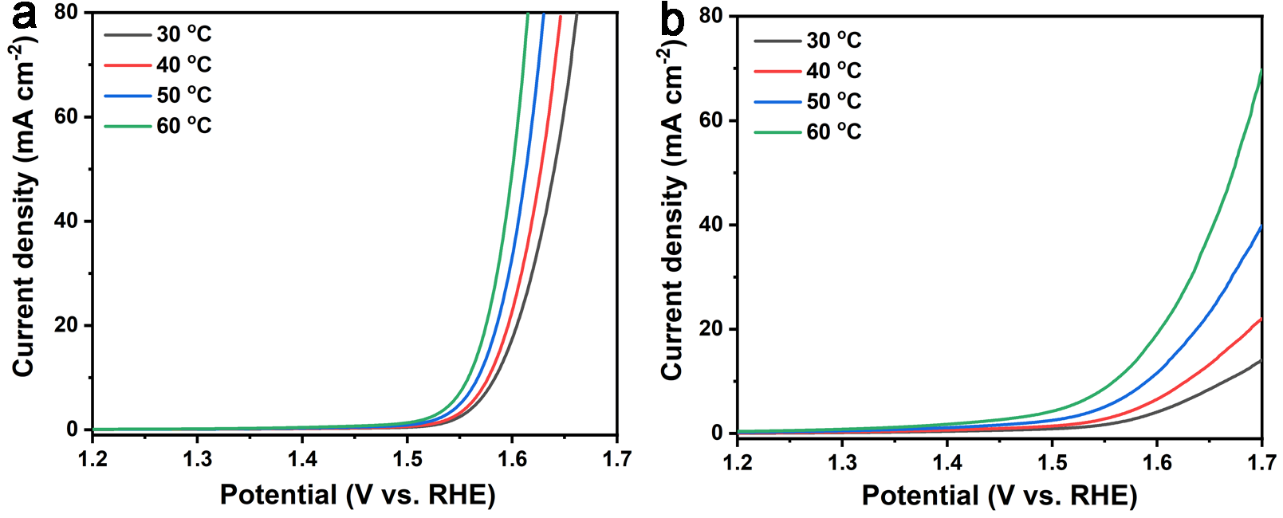


**Supplementary Figure 15**. LSV curves of (a) Co SA-NDGs and (b) NDGs measured at different temperature.

The electrochemical activation energy (*E_a_*) of OER can be estimated by the Arrhenius relationship:

$\text{L}\text{og}\text{j}\text{ }\text{=}\text{ }\frac{\text{-}\text{E}_{\text{a}}}{\text{ln10*}\text{R}\text{*}\text{T}}\text{ }\text{+}\text{ }\text{cons}\text{tan}\text{t}$ (4)

where *j* is the current density at the overpotential of 350 mV, *R* is the universal gas constant (8.314 J K^-1^ mol^-1^), *T* is the temperature. The *E_a_* could be calculated from the slope of the fitted Arrhenius plot.


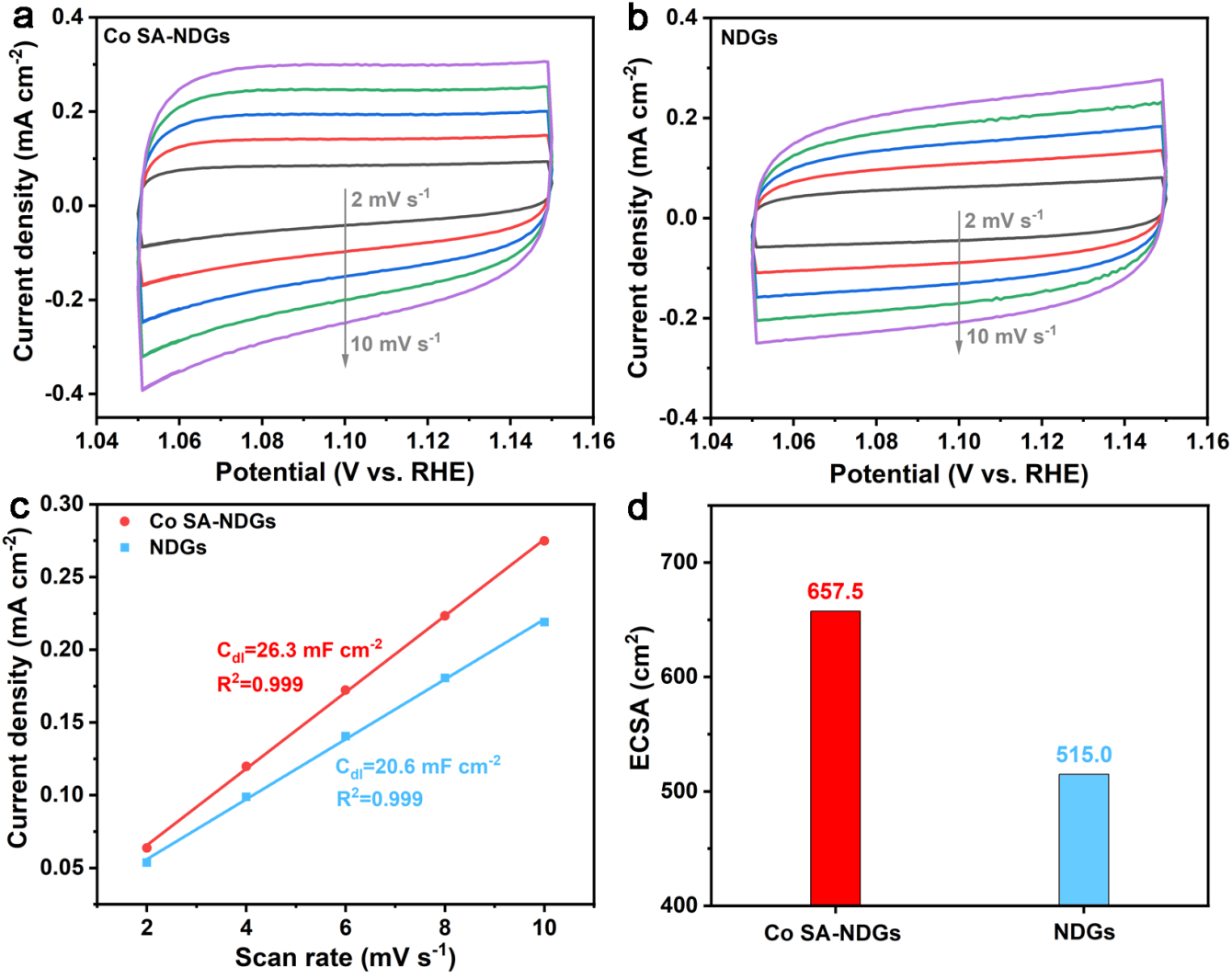


**Supplementary Figure 16**. CV curves of (a) Co SA-NDGs and (b) NDGs at 2, 4, 6, 8, and 10 mV s^-1^. (c) The capacitive current measured at 1.10 V plotted as a function of scan rate. (d) Comparison of ECSA of Co SA-NDGs and NDGs.

A specific capacitance of 1 cm^2^ flat surface-area (*C_s_*) generally corresponds to 0.04 mF cm^-2^ According to the reported work^2^. Therefore, the ECSA can be obtained from the following equation:

$\text{ECSA}\text{ }\text{=}\text{ }\frac{\text{C}_{\text{dl}}}{\text{0.04}}\text{ }$ (5)


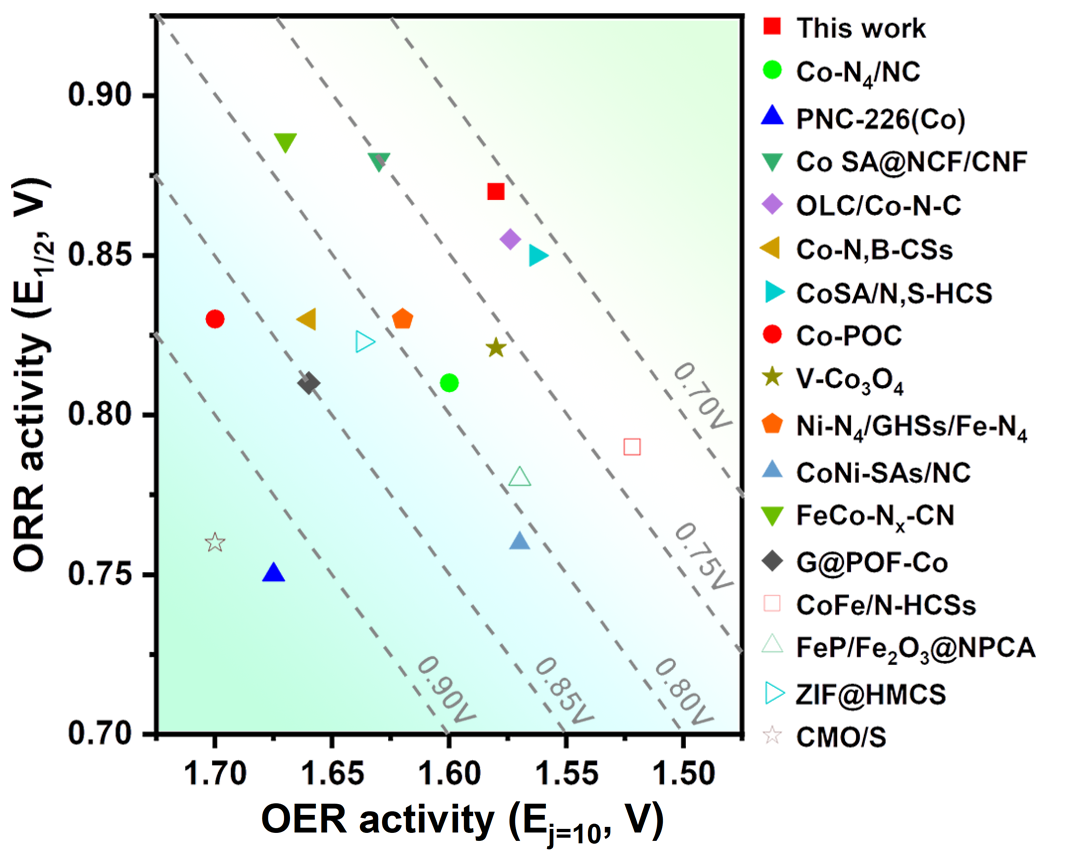


**Supplementary Figure 17**. Comparison of bifunctional ORR/OER activities of Co SA-NDGs and other non-precious metal catalysts reported.


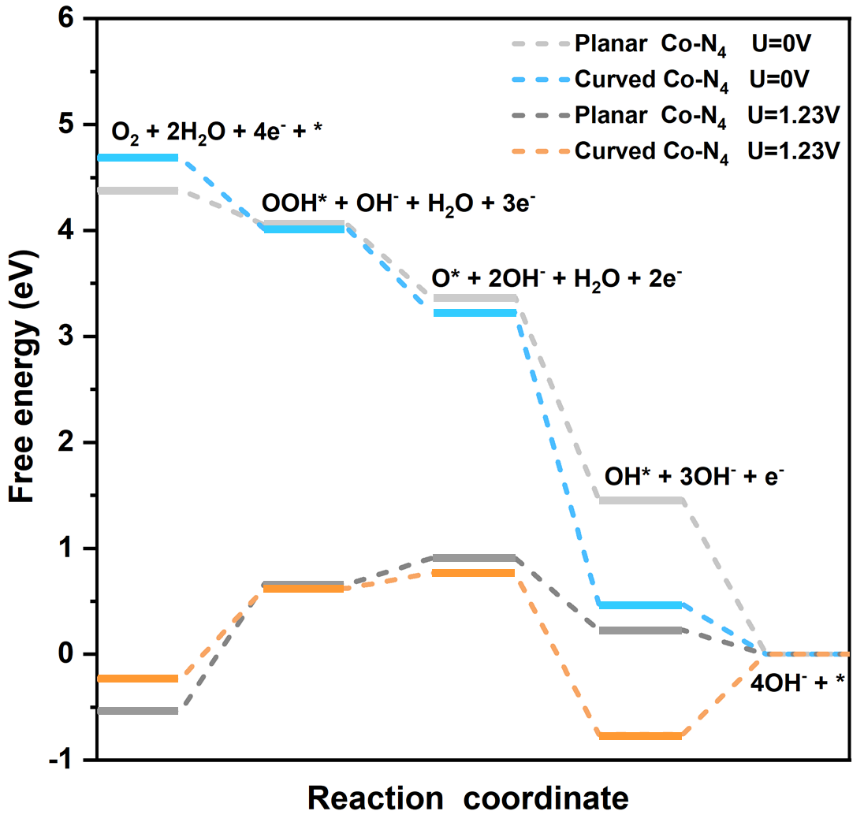


**Supplementary Figure 18**. Free energy diagram of the ORR on planar Co-N_4_ site and curved Co-N_4_ site in alkaline media.


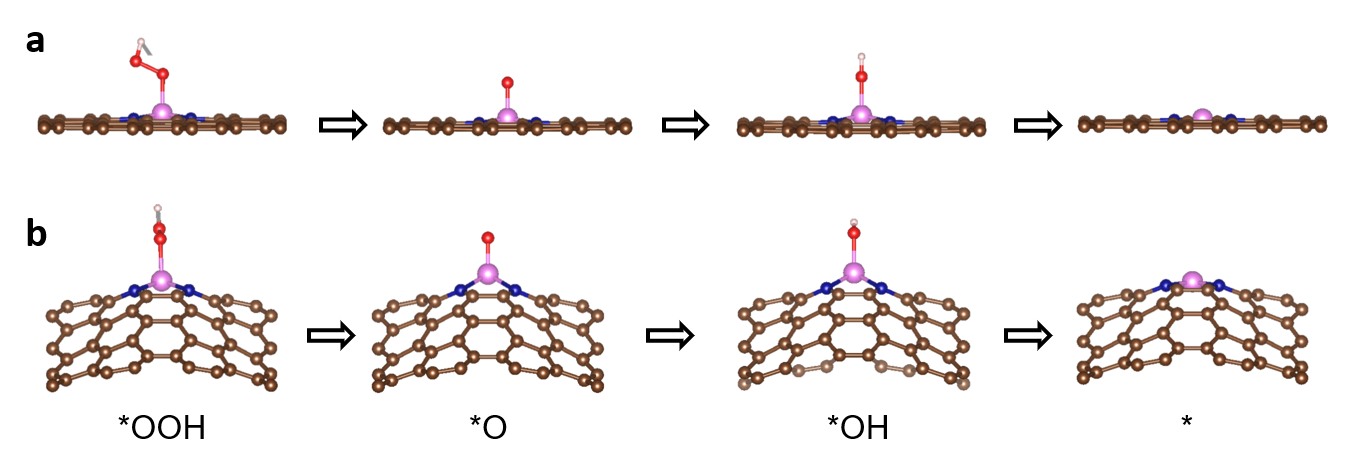


**Supplementary Figure 19**. The optimized intermediate species along the reaction pathway of ORR on (a) the planar Co-N_4_ and (b) the curved Co-N_4_.

**
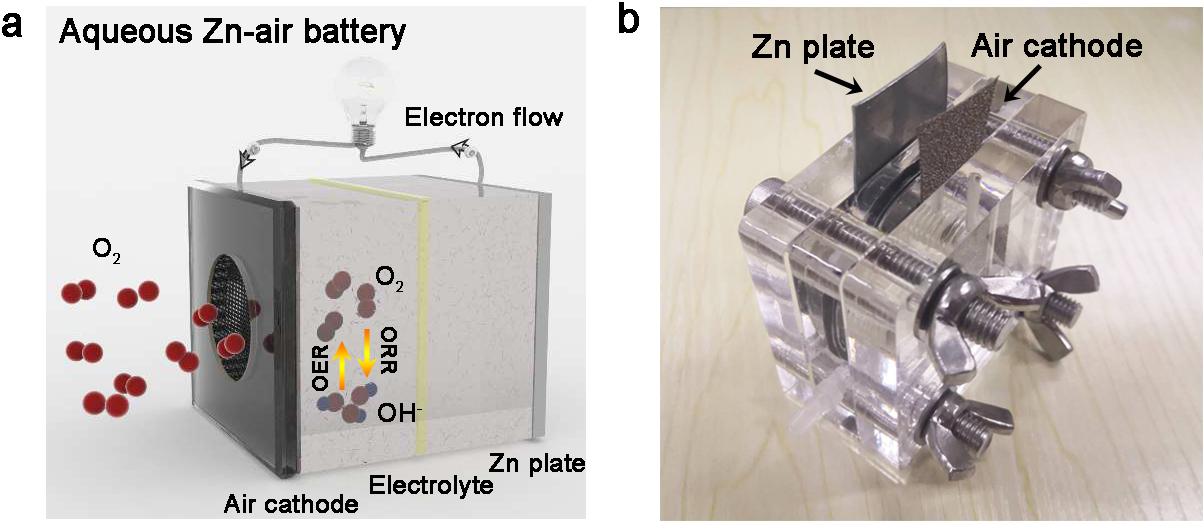
**

**Supplementary Figure 20**. (a) Schematic illustration of aqueous ZABs. (b) Optical photograph of the assembled ZABs.

**
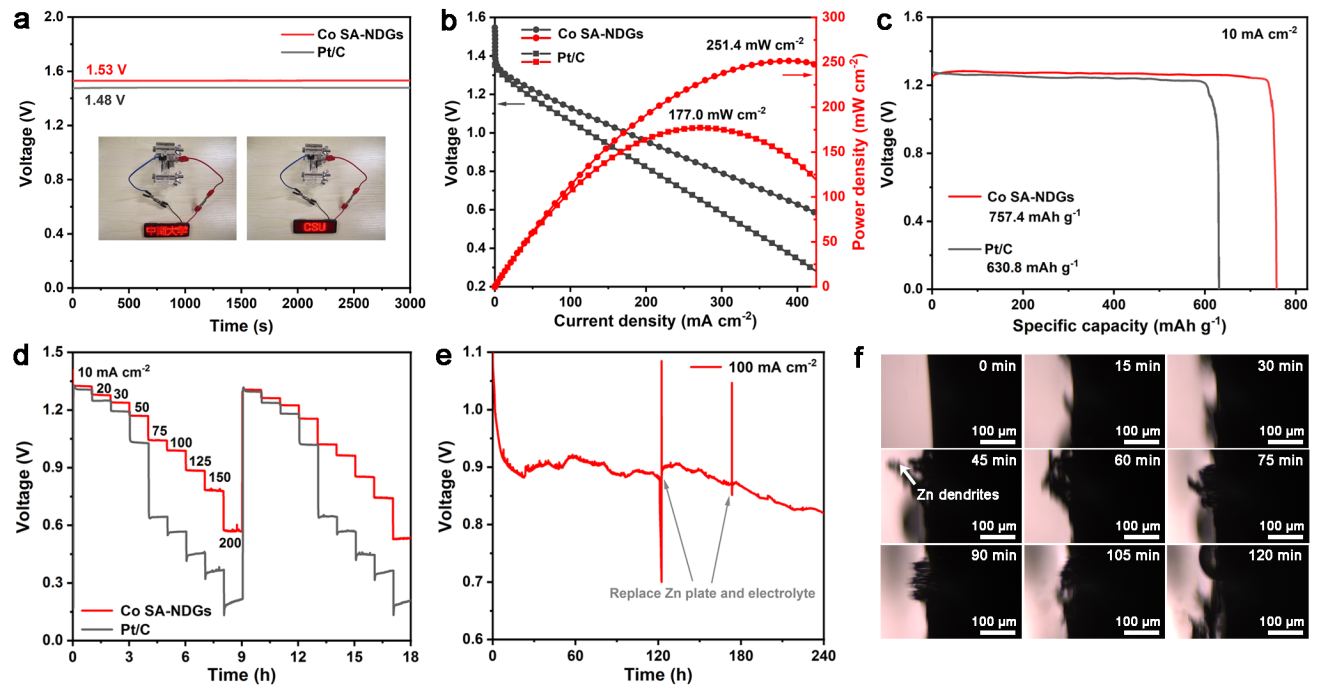
**

**Supplementary Figure 21**. (a) OCV curves of the aqueous ZABs with Co SA-NDGs and Pt/C. Inset shows the photograph of practical application of the aqueous ZABs. (b) Discharge curves and corresponding power density curves of aqueous ZABs using Co SA-NDGs and Pt/C. (c) Specific capacities at 10 mA cm^-2^. (d) Rate performance. (e) Discharge curves of the aqueous ZABs with Co SA-NDGs at 100 mA cm^-2^. (f) In situ optical visualization observations of the interface change of the Zn|electrolyte under the discharge at 20 mA cm^-2^ in 6 M KOH + 0.2 M Zn(Ac)_2_ electrolyte.


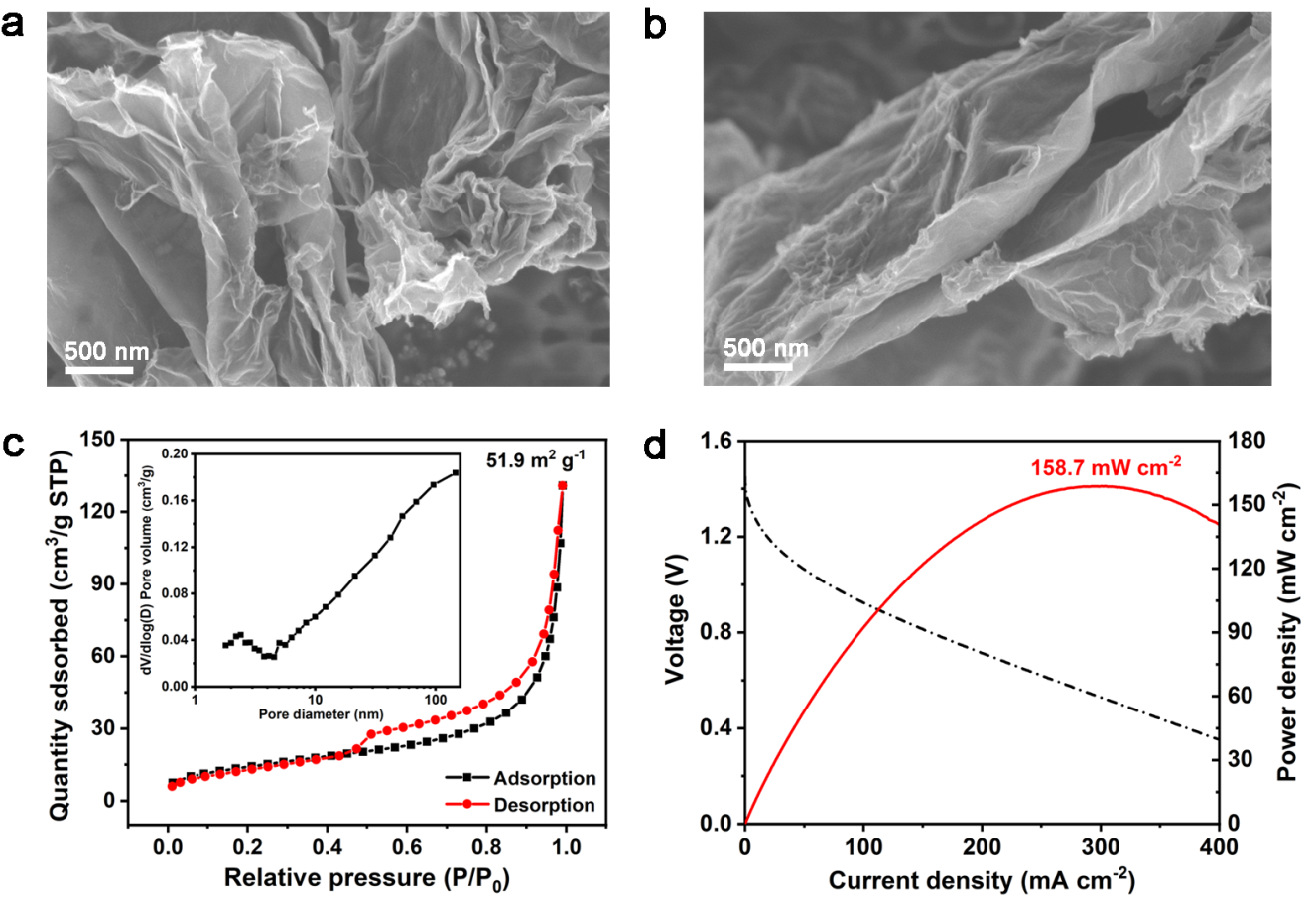


**Supplementary Figure 22**. (a, b) SEM images, (c) N_2_ adsorption/desorption isotherms of the control Co SA-NDGs. Inset shows the corresponding pore size distributions. (d) Galvanostatic discharge curve and the corresponding power density curve of the aqueous ZABs with the control Co SA-NDGs. Noted: the control Co SA-NDGs was prepared by the same procedure as that used for synthesis of Co SA-NDGs except without freeze-drying treatment.


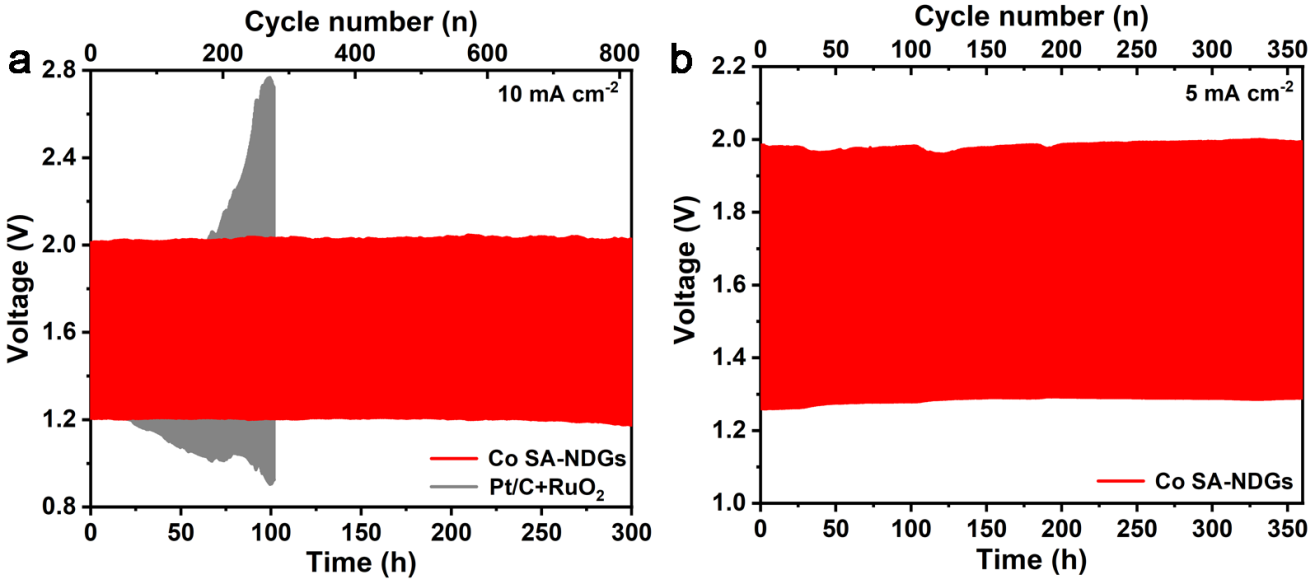


**Supplementary Figure 23**. Charging/discharging cycling performance of aqueous ZABs at current density of (a) 10 mA cm^-2^ (each cycle of 22 min) and (b) 5 mA cm^-2^ (each cycle of 1 h).

**
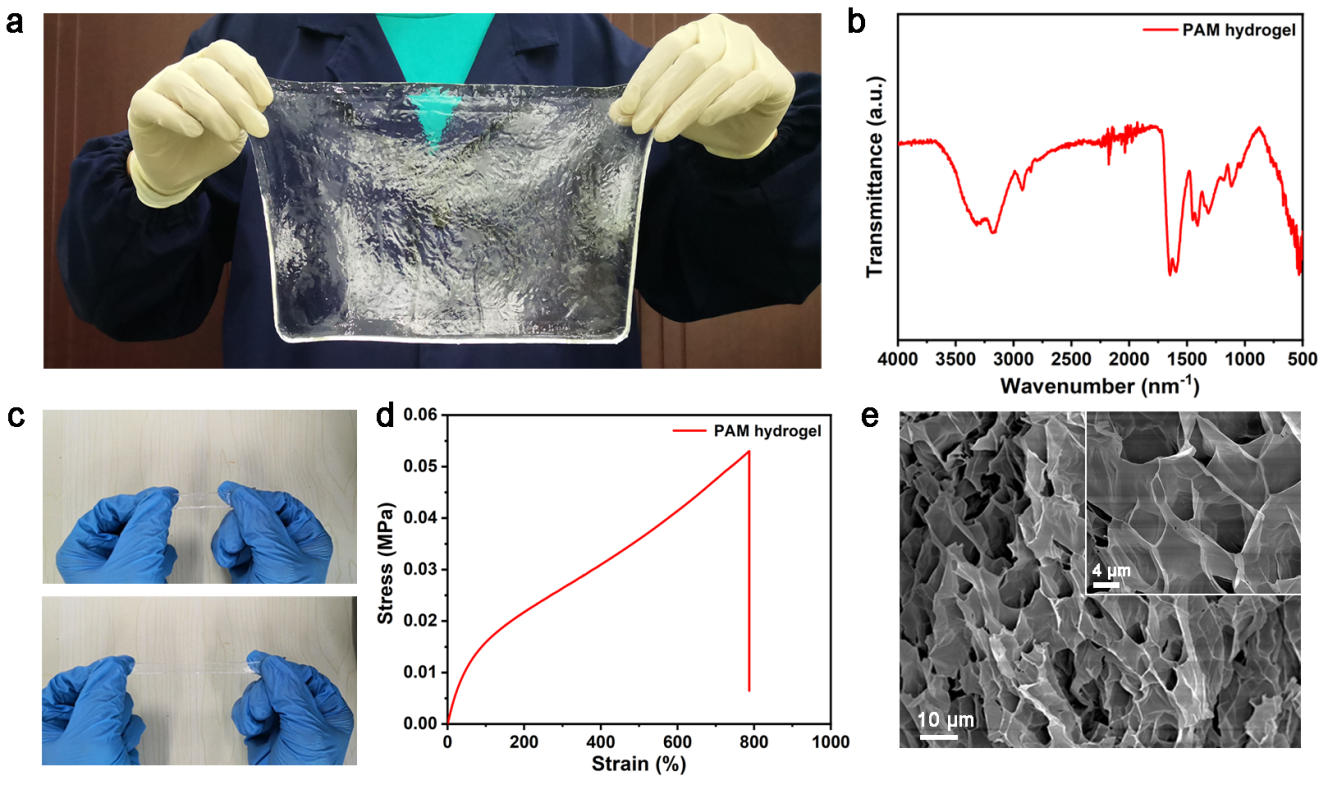
 Supplementary Figure 24.** (a) Photograph of the amplification-synthesized PAM hydrogel. (b) Fourier transform infrared spectrum of the PAM hydrogel. (c) Photographs of the PAM hydrogel in stretched state. (d) Tensile stress-strain curve of PAM hydrogel. (e) SEM images of the freeze-dried PAM hydrogel.

The stretchable PAM hydrogel electrolyte by free radical polymerization approach, followed by soaking in 6 M KOH + 0.2 M Zn(Ac)_2_ solution to absorb significant salt electrolyte via solvent exchange. It is noted that this method can amplify production. The PAM chains form porous network structure via covalent cross-links and hydrogen bonds. Several absorption bands (e.g., N-H stretching/bending vibration and C-O stretching vibration) are detected in Fourier transform infrared spectrum^3^. The PAM hydrogel displays a good tensile strength and excellent stretchability (up to 780%).


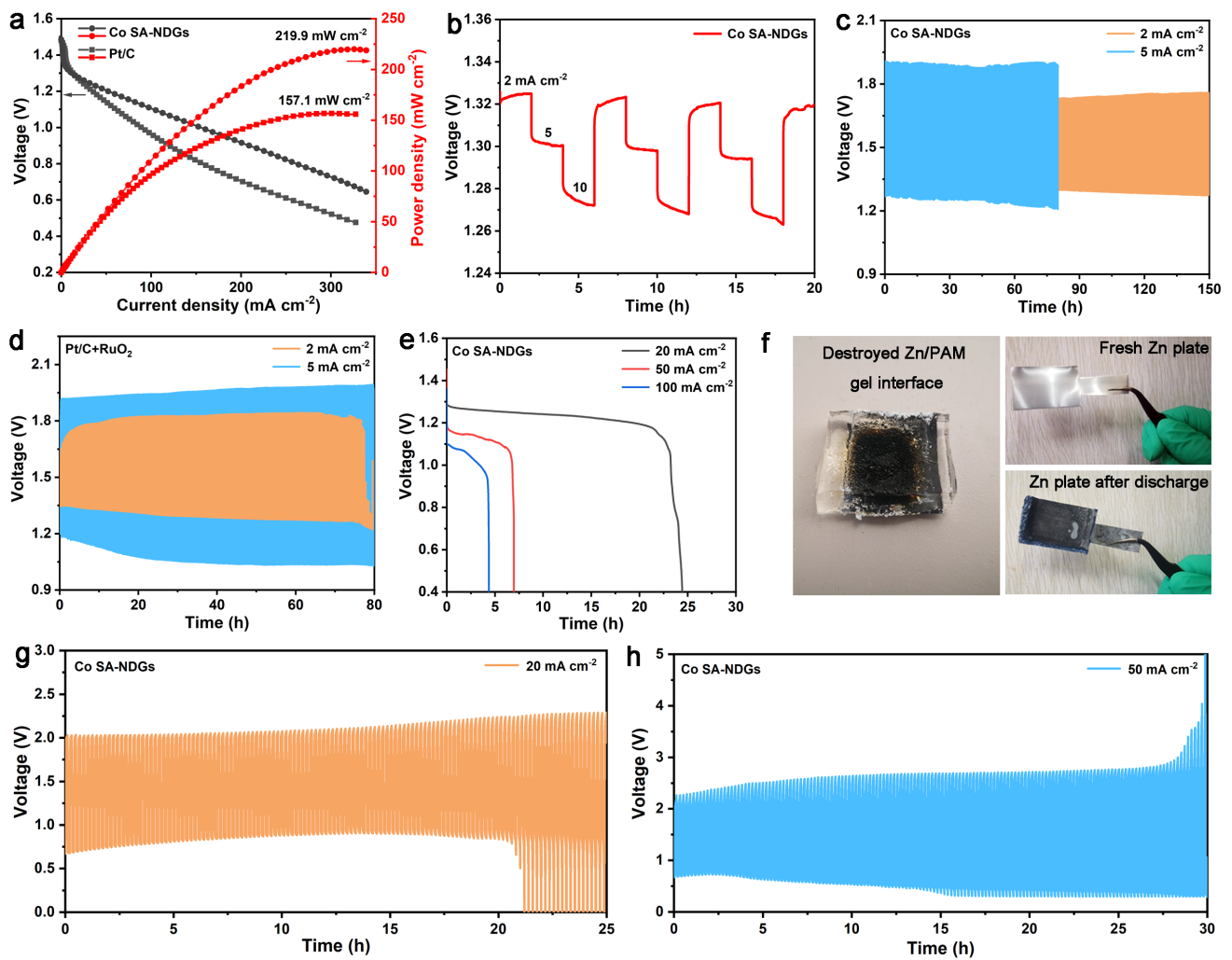


**Supplementary Figure 25.** (a) Galvanostatic discharge curves and the corresponding power density curves of the quasi-solid-state ZABs using PAM hydrogel electrolyte. (b) Rate performance. Charging/discharging performance of quasi-solid-state ZABs with (c) Co SA-NDGs and (d) Pt/C+RuO_2_. (e) Galvanostatic discharge voltage platforms of the quasi-solid-state ZABs with Co SA-NDGs. (f) Photographs of PAM hydrogel electrolyte and Zn plate after discharge. Charging/discharging cycling performance of the Co SA-NDGs-based quasi-solid-state ZABs at (g) 20 mA cm^-2^ and (h) 50 mA cm^-2^.

The charging/discharging plateaus with the small overpotential are recorded at 2 and 5 mA cm^-2^ for the quasi-solid-state ZABs with Co SA-NDGs, by contrast to that of quasi-solid-state Pt/C+RuO_2_-based ZABs, validating the excellent reversibility and robustness. The main component of Zn dendrites after cycling test is identified as ZnO (Supplementary Fig. 26)^4^.


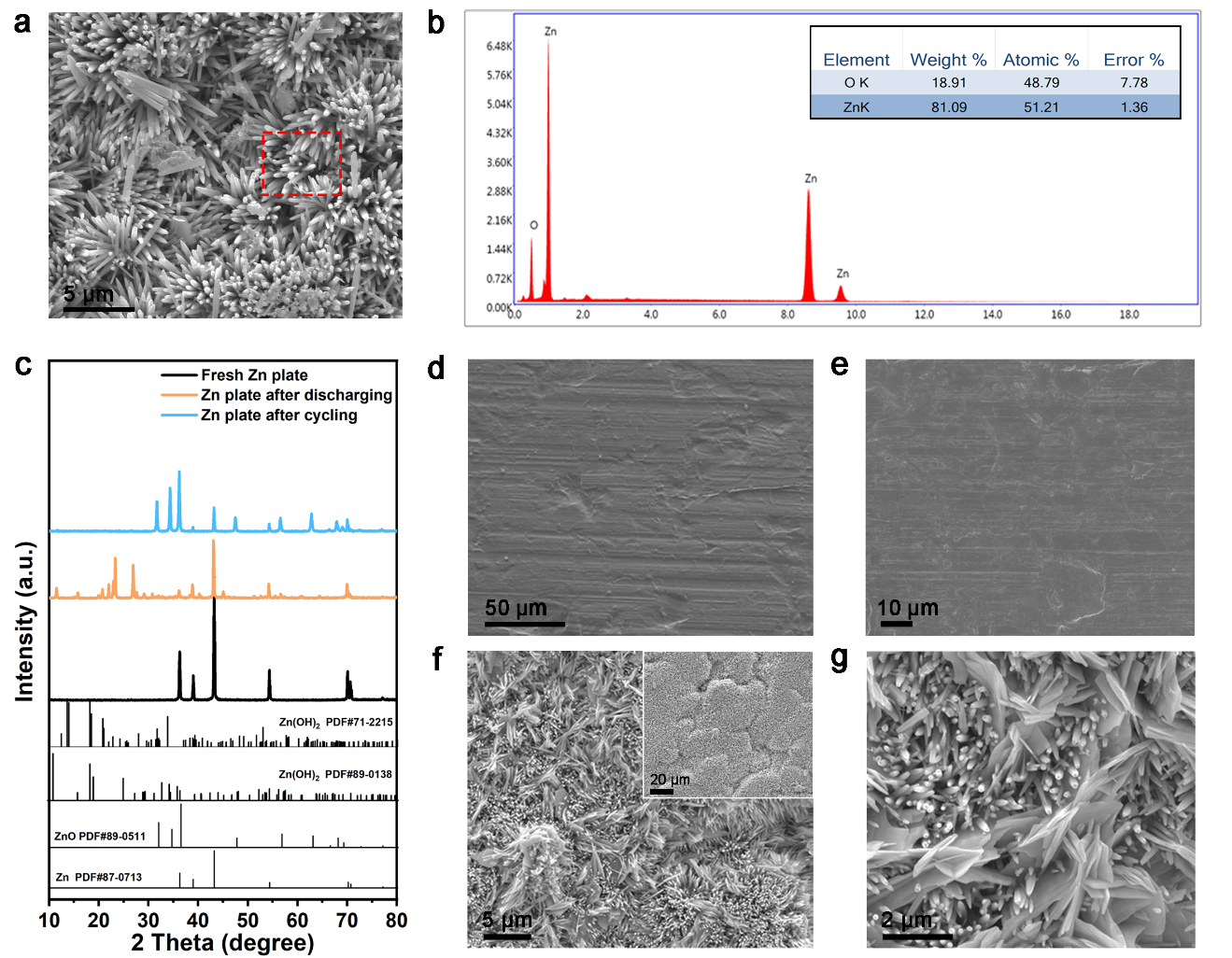


**Supplementary Figure 26**. (a) SEM image of Zn anode after cycling test. (b) The corresponding EDS results of the cycled Zn plate. (c) XRD patterns of various Zn samples. It suggests that the discharging and charging/discharging products are Zn(OH)_2_ and ZnO, respectively. (d, e) SEM images of fresh Zn plate. (f, g) SEM images of Zn plate after discharging.


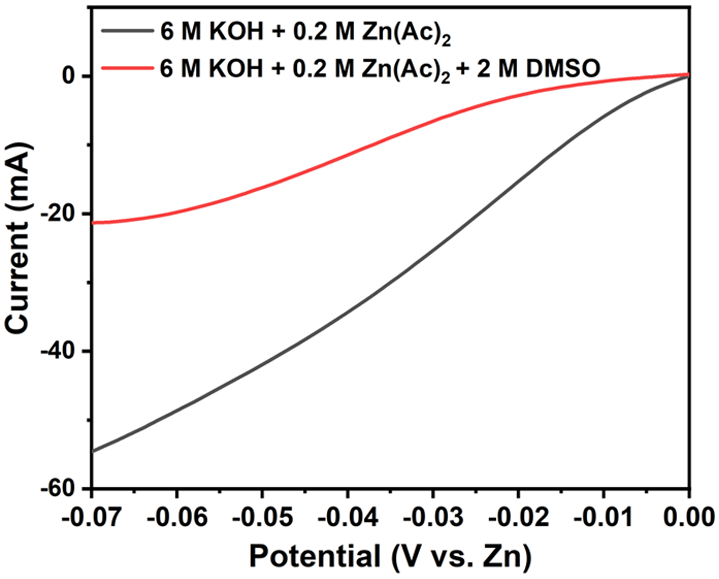


**Supplementary Figure 27**. Hydrogen evolution reaction activity in different electrolyte.


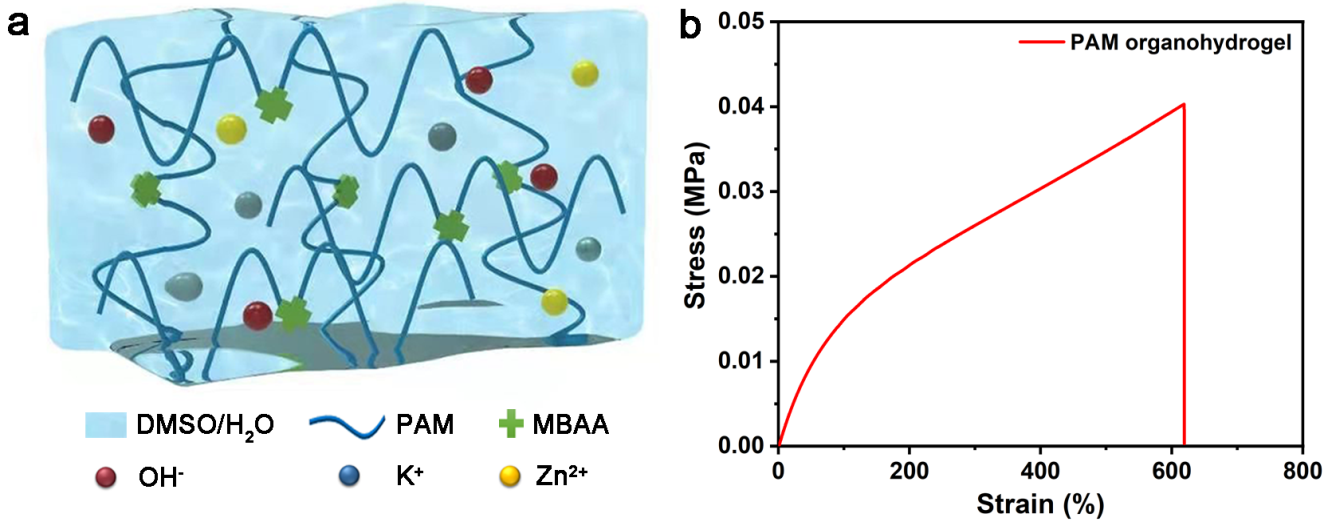


**Supplementary Figure 28.** (a) Schematic illustration of PAM organohydrogel electrolyte synthesized in binary solvent systems of DMSO/H_2_O. (b) Tensile stress-strain curve of PAM organohydrogel.


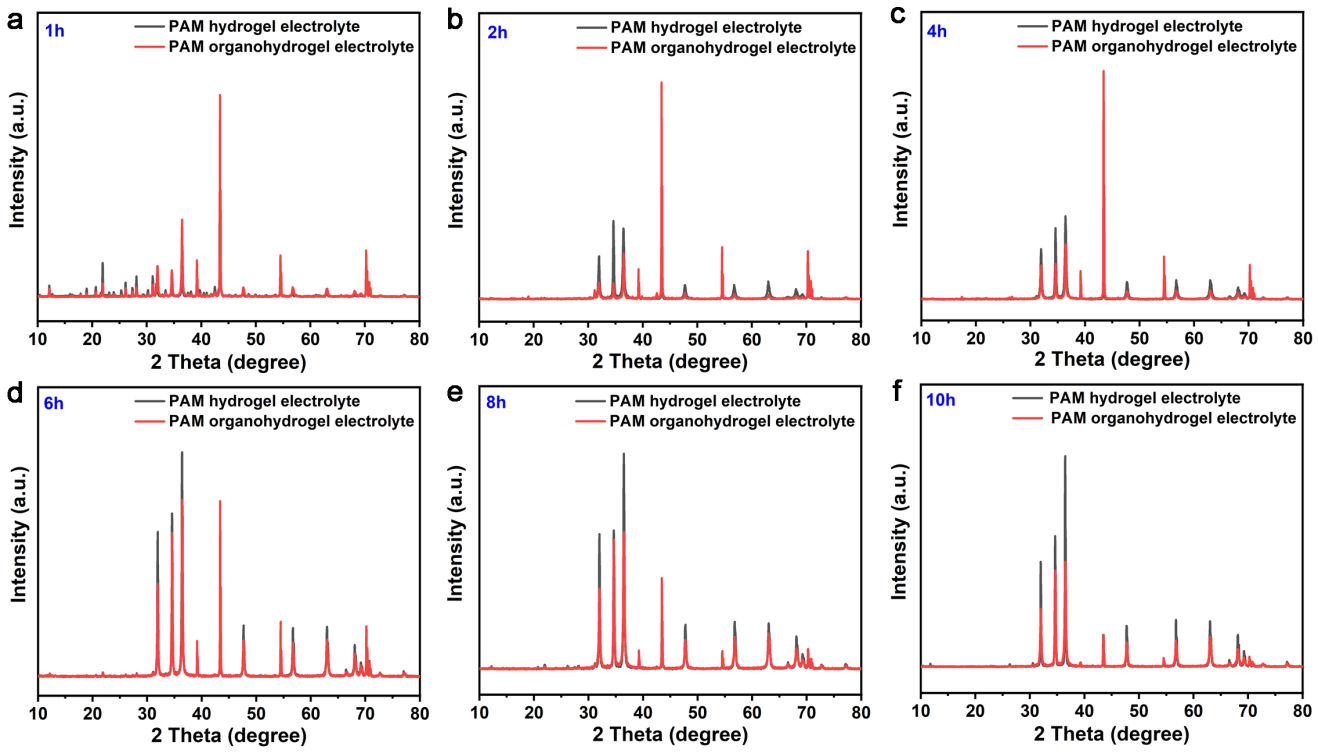


**Supplementary Figure 29.** Ex situ XRD patterns of Zn plates after cycling tests under the different charging-discharging time at 20 mA cm^-2^: (a) 1 h, (b) 2 h, (c) 4 h, (d) 6 h, (e) 8 h, and (f) 10 h.


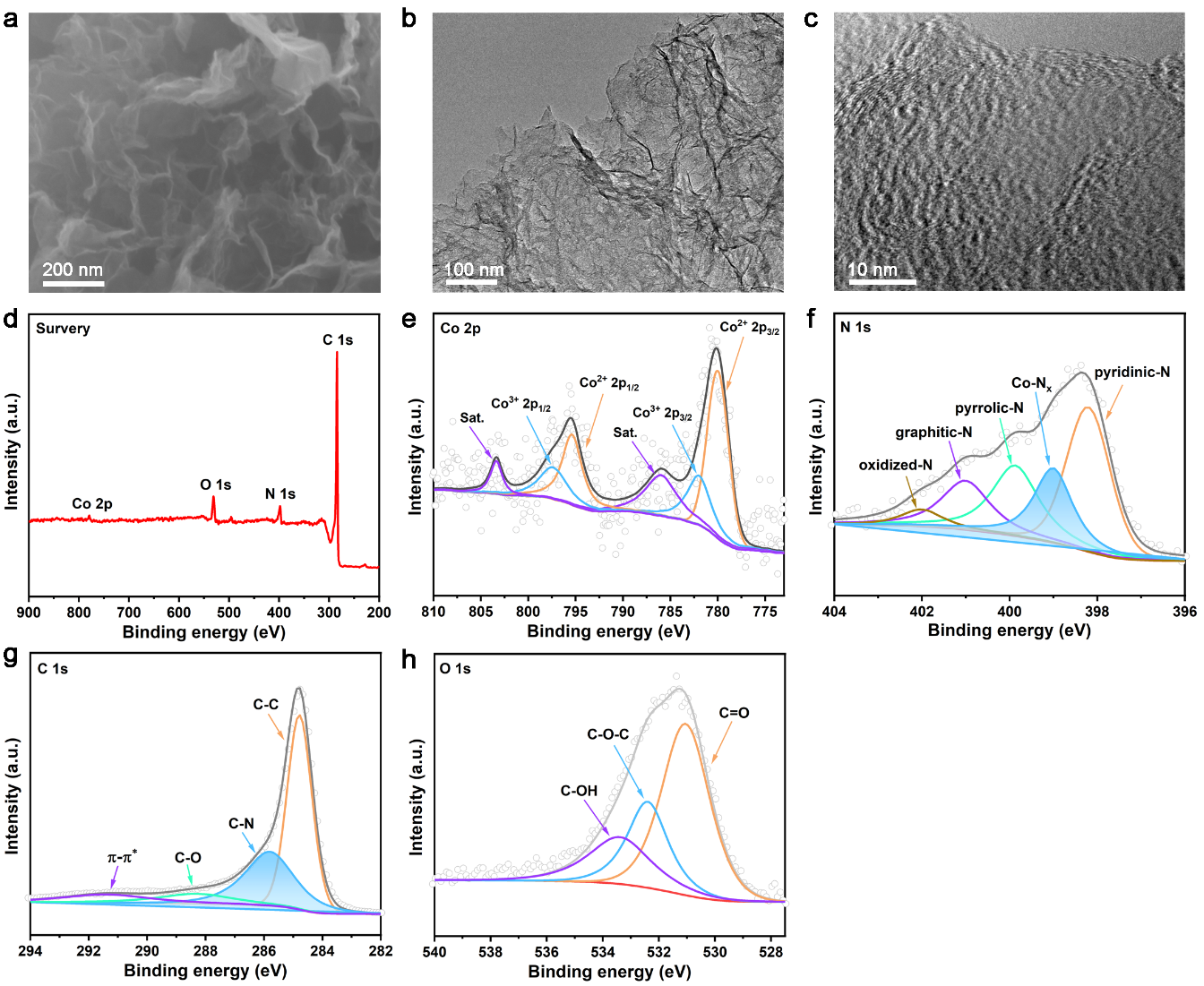


**Supplementary Figure 30.** (a) SEM image and (b) TEM image for Co SA-NDGs after 50 h of cycling test at 100 mA cm^-2^. It is noted that the Co SA-NDGs-based air cathode is harvested after cycling test for further characterizations.


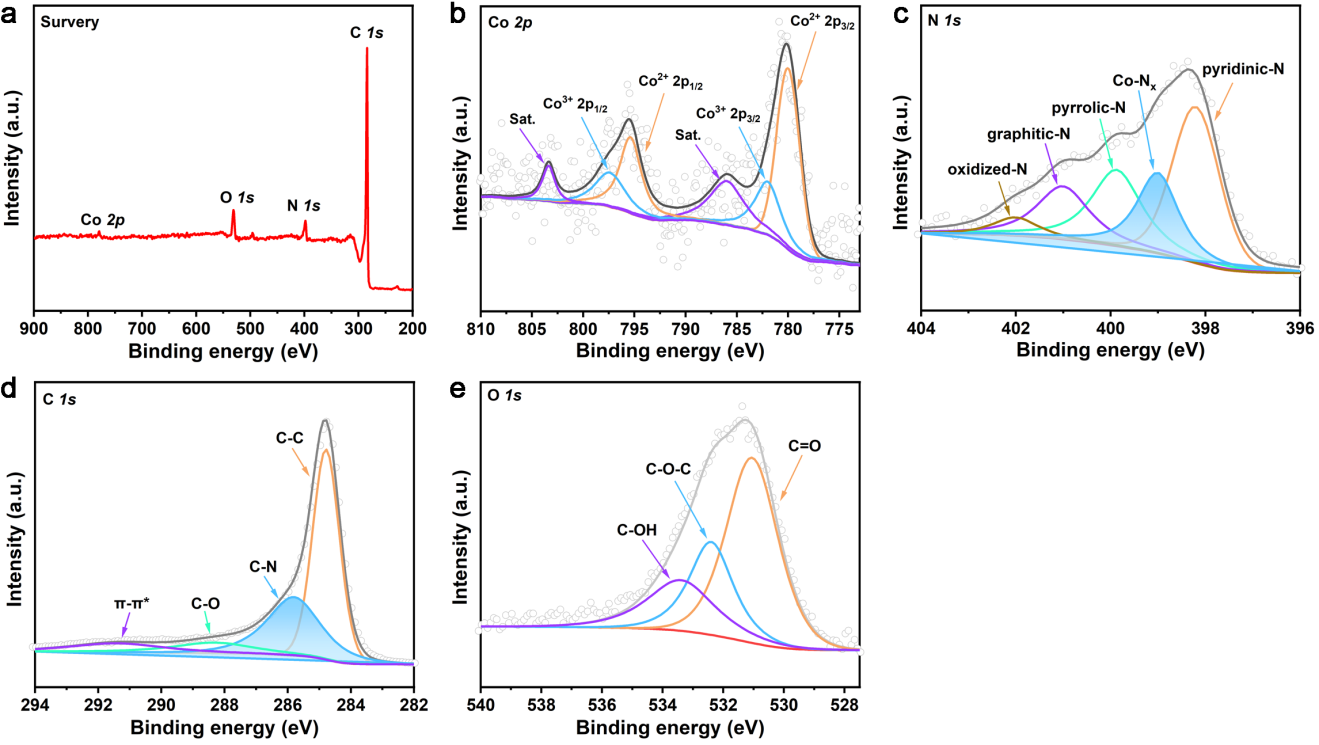


**Supplementary Figure 31.** (a) XPS survey spectrum, (b) Co *2p* spectrum, (c) N *1s* spectrum, (d) C *1s* spectrum and (e) O *1s* spectrum for Co SA-NDGs after 50 h of cycling test at 100 mA cm^-2^.


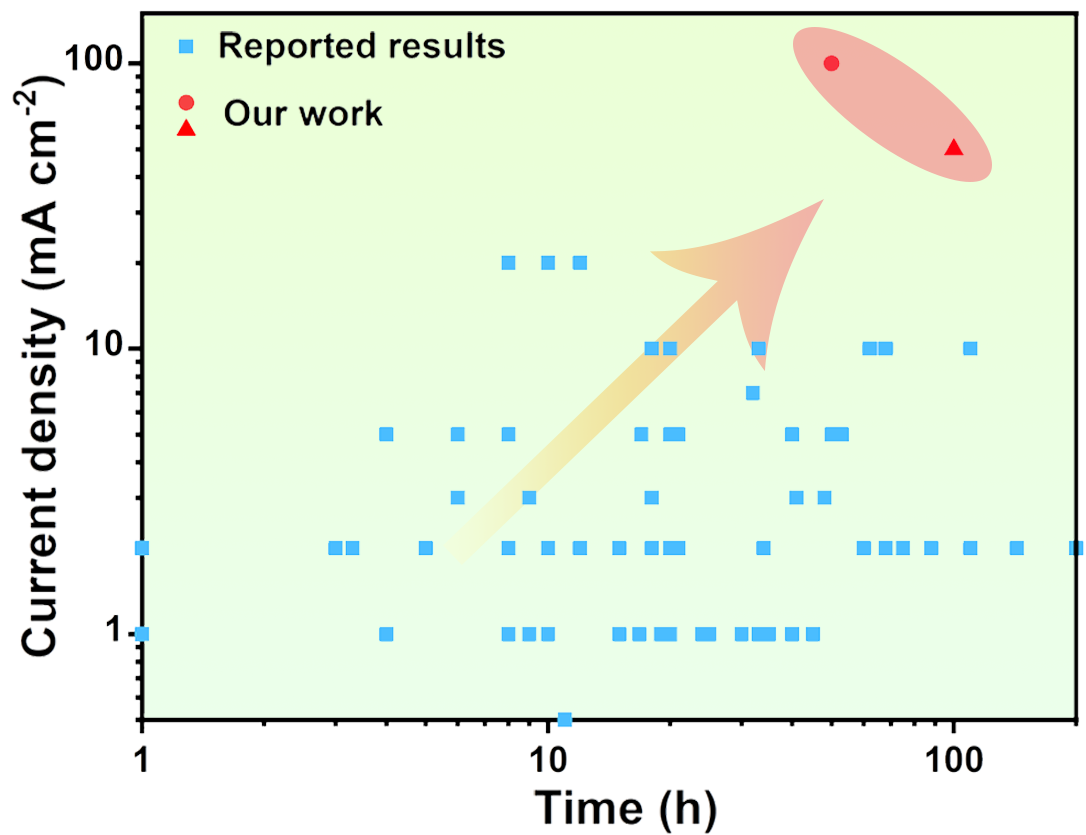


**Supplementary Figure 32.** Data analysis of cycling performance distribution for the reported quasi-solid-state ZABs measured at 25 °C.


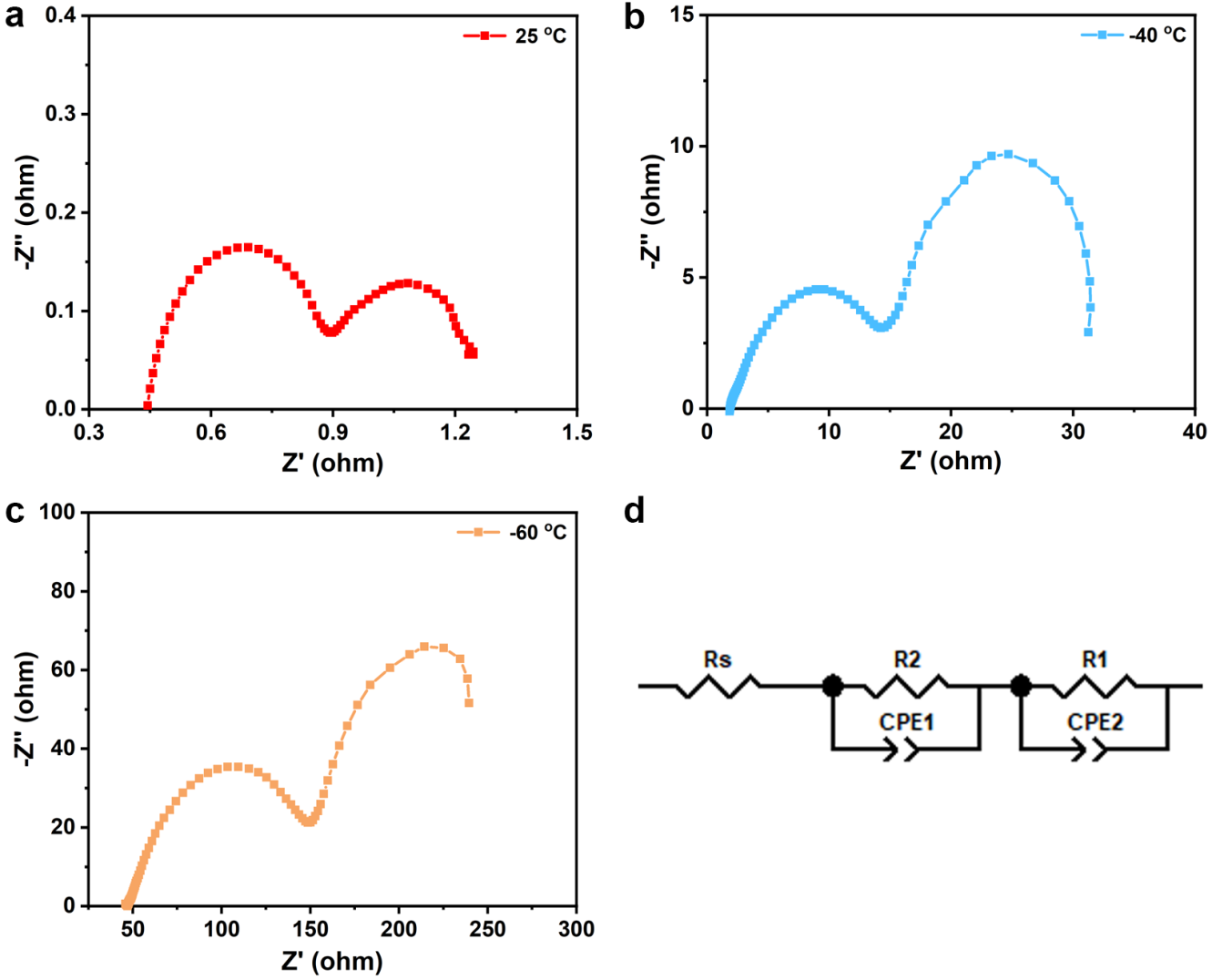


**Supplementary Figure 33.** EIS results of the quasi-solid-state ZABs with Co SA-NDGs measured at (a) 25 ^o^C, (b) -40 ^o^C and (c) -60 ^o^C. (d) Equivalent circuit diagram.


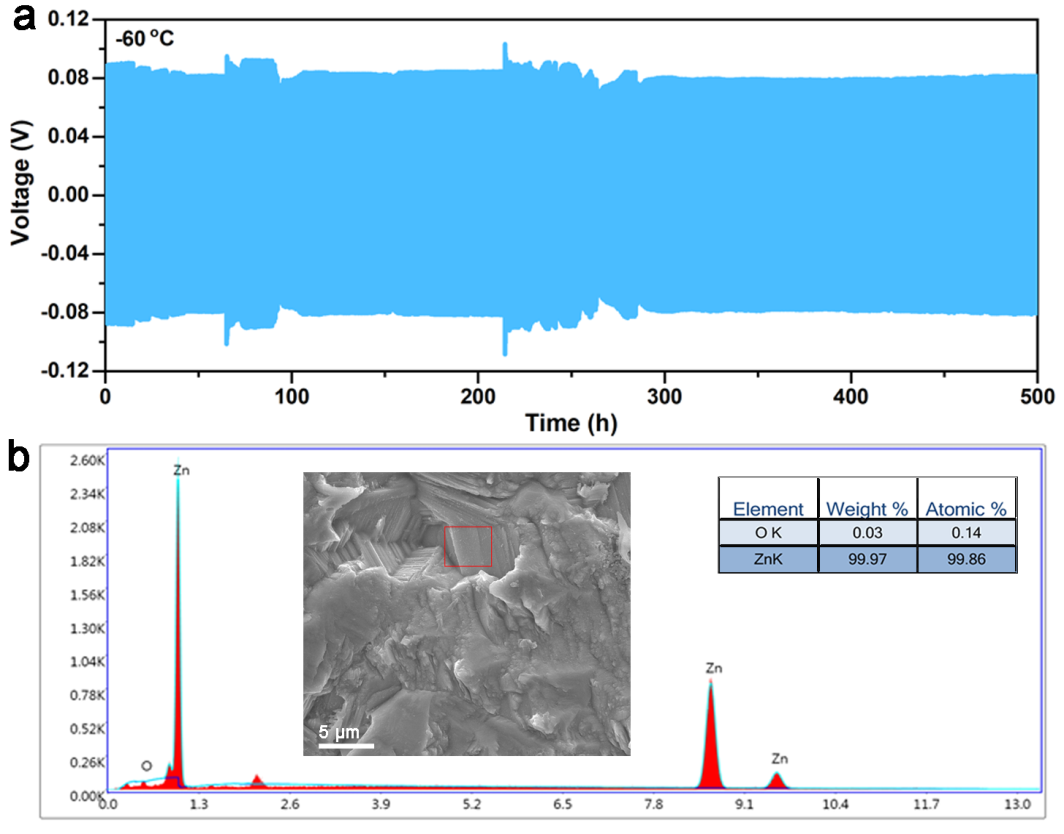


**Supplementary** **Figure 34.** (a) Cycling performance (@0.5 mA cm^-2^) of the symmetric Zn||Zn cell employing the PAM organohydrogel electrolyte at -60 ^o^C. (b) EDS result of Zn plate after 500 h cycling test. Inset shows the corresponding SEM image and atomic ratio.


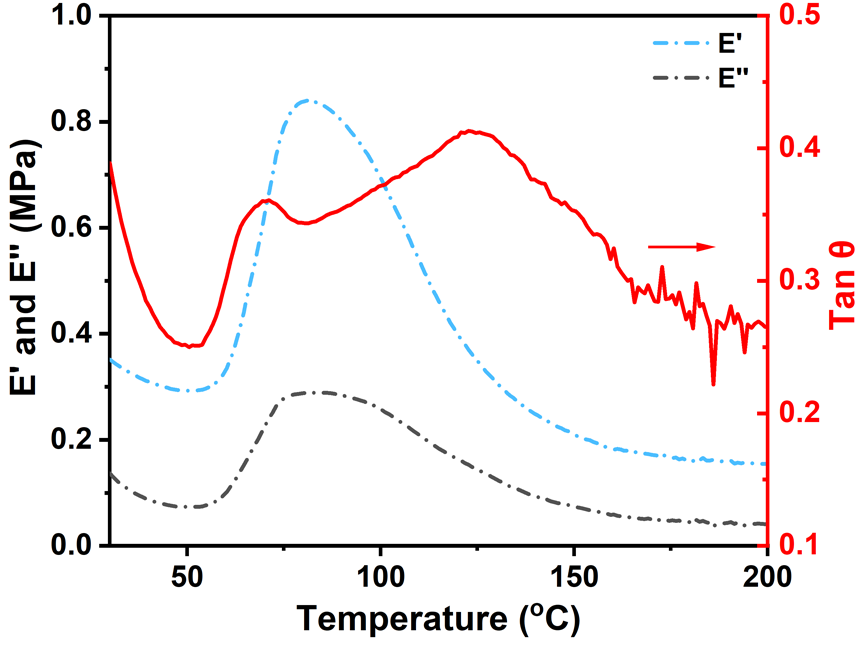


**Supplementary Figure 35**. DMA analysis of PAM organohydrogel under temperature window ranging from 25 to 200 ^o^C.


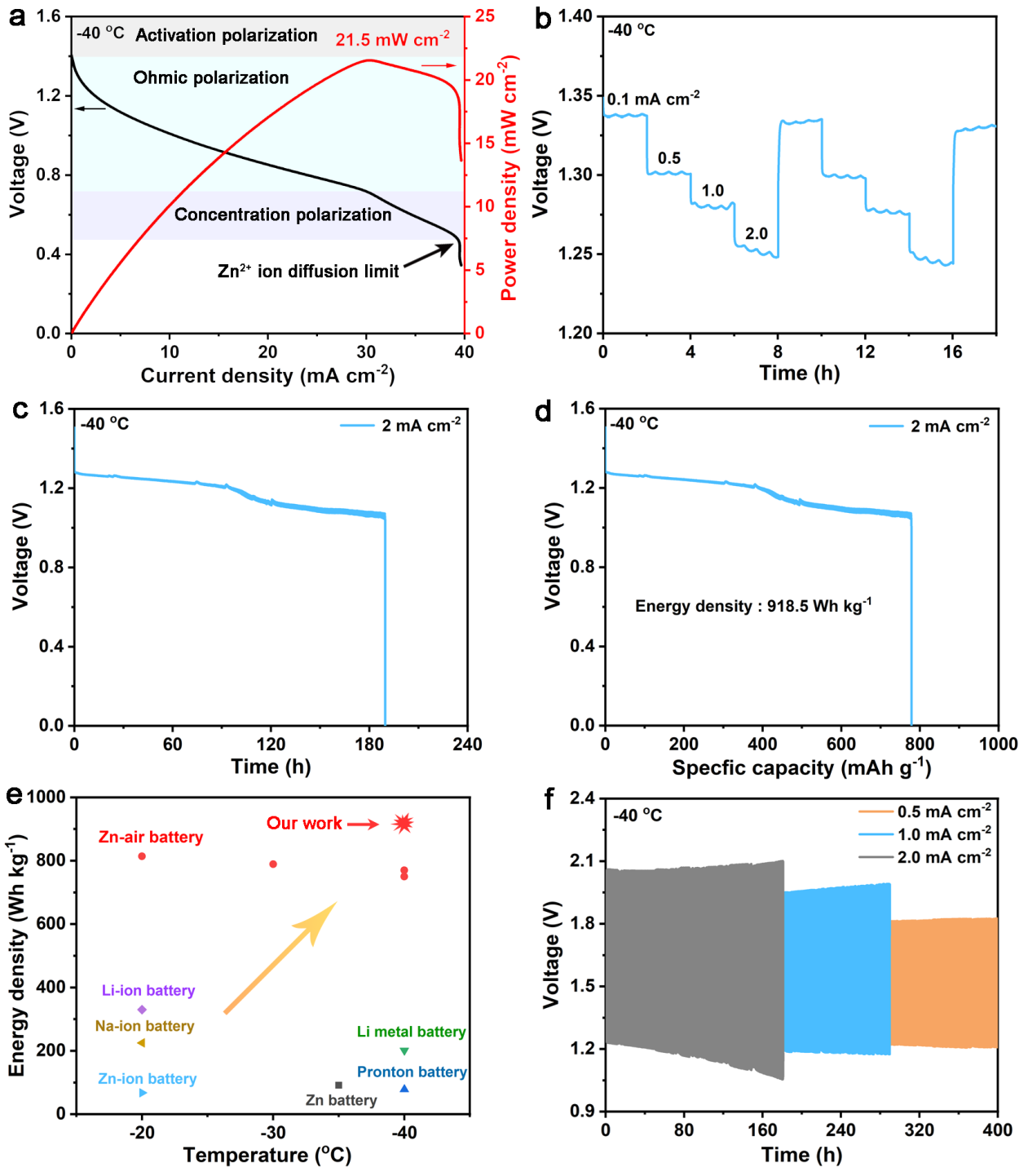


**Supplementary Figure 36**. (a) Galvanostatic discharge curves and the corresponding power density curves of the quasi-solid-state ZABs with Co SA-NDGs at -40 ^o^C. (b) Rate performance of the Co SA-NDGs-based quasi-solid-state ZABs. (c) Galvanostatic discharge voltage platform and (d) specific capacity of the quasi-solid-state ZABs with Co SA-NDGs at 2 mA cm^-2^. (e) Ragone plots for assembled quasi-solid-state ZABs’ energy density and operating temperature with reported low-temperature solid-state batteries previously reported. (f) Charging/discharging cycling performance of quasi-solid-state ZABs with Co SA-NDGs at -40 ^o^C.

As shown in Supplementary Fig. 36a, the dominant factor of discharge behavior changes from ohmic polarization to concentration polarization with the increase of voltage at -40 °C. The quasi-solid-state ZABs using Co SA-NDGs delivers a maximum power density of 21.5 mW cm^-2^. Therefore, a large voltage driving force is required in ultra-low-temperature environment to reach similar current density level measured in ambient condition^5,6^. Supplementary Fig. 36b shows the rate performance of quasi-solid-state ZABs with Co SA-NDGs at -40 °C. After twice current density fluctuation tests, the discharging voltage hardly decays. The excellent charging/discharging cycling performance with high-capacity retention over 90% is recorded at different current density in Supplementary Fig. 36f.


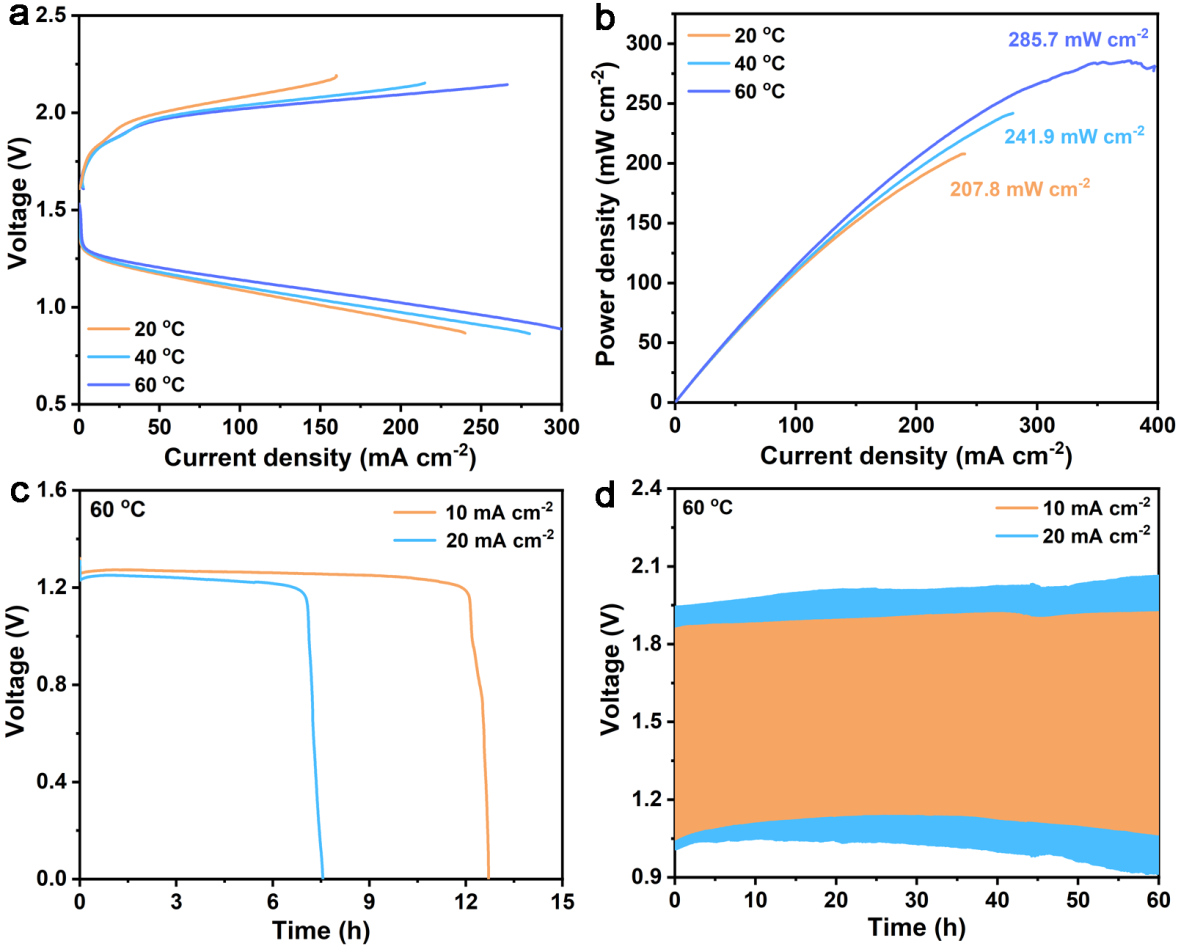


**Supplementary Figure 37**. (a) Charging/discharging curves and (b) corresponding power density curves of quasi-solid-state ZABs with Co SA-NDGs at 20, 40 and 60 ^o^C. (c) Galvanostatic discharge voltage platforms at 10 and 20 mA cm^-2^. (d) Charging/discharging cycling performance of quasi-solid-state ZABs with Co SA-NDGs measured at 60 ^o^C.

**Supplementary Table 1.** EXAFS fitting parameters at the Co K-edge.

| Samples | Shell | N ^a^ | R (Å) ^b^ | σ^2^ (Å^2^·10^-3^) ^c^ | ΔE_0_ (eV) ^d^ | *R* factor (%) |
| --- | --- | --- | --- | --- | --- | --- |
| Co SA-NDGs | Co-N | 3.3 | 1.85 | 6.2 | -8.8 | 0.001 |
|  | Co-C | 7.2 | 2.51 | 6.6 | -8.8 |  |

*^a^* *N*: coordination numbers; *^b^* *R*: bond distance; *^c^* *σ*^2^: Debye-Waller factors; *^d^* Δ*E*_0_: the inner potential correction. *R* factor: goodness of fit.

**Supplementary Table 2.** Performance comparison of bifunctional oxygen catalysts.

| Catalysts | E_onset_  (V) | E_1/2_  (V) | E_j=10_ (V) | △E  (V) | Reference |
| --- | --- | --- | --- | --- | --- |
| Co SA-NDGs | 1.03 | 0.87 | 1.58 | 0.71 | This work |
| CNT@CoSA-Co/NCP | 1.00 | 0.87 | 1.61 | 0.74 | *Adv. Funct. Mater.*  **2021**, *31*, 2103360. |
| NGM-Co | 0.92 | 0.80 | 1.75 | 0.95 | *Adv. Mater.*  **2017**, *29*, 1703185. |
| H-Co@FeCo/N/C | 1.03 | 0.91 | 1.61 | 0.70 | *Appl. Catal. B: Environ.*  **2020**, *278*, 119259. |
| Fe/Ni-N_x_/OC | 1.05 | 0.94 | 1.67 | 0.73 | *Adv. Mater.*  **2020**, *32*, 2004670. |
| Fe/N-G-SAC | 0.99 | 0.89 | 1.60 | 0.71 | *Adv. Mater.*  **2020**, *32*, 2004900. |
| Co-N_4_/NC | 0.93 | 0.81 | 1.60 | 0.79 | *Nano-Micro Lett.*  **2021**, *13*, 60. |
| FeN_x_-PNC | 0.997 | 0.85 | 1.63 | 0.78 | *ACS Nano*  **2018**, *12*, 1949. |
| Fe-NC SAC | 1.02 | 0.88 | 1.68 | 0.80 | *J. Mater. Chem. A*  **2020**, *8*, 9981. |
| Co SA@NCF/CNF | 1.01 | 0.88 | 1.63 | 0.75 | *Adv. Mater.*  **2019**, *31*, 1808267. |
| Co-C_3_N_4_/CNT | 0.9 | 0.85 | 1.61 | 0.76 | *J. Am. Chem. Soc.*  **2017**, *139*, 3336. |
| S,N-Fe/N/C-CNT | 0.97 | 0.85 | 1.60 | 0.75 | *Angew. Chem. Int. Ed.*  **2017**, *56*, 610. |
| CoN_4_/NG | 0.98 | 0.87 | 1.61 | 0.74 | *Nano Energy*  **2018**, *50*, 691. |
| CoSA/N,S-HCS | 0.96 | 0.85 | 1.56 | 0.71 | *Adv. Energy Mater.*  **2020**, *10*, 2002896. |
| UNT Co SAs/N-C | 0.97 | 0.89 | 1.61 | 0.72 | *Nano Energy*  **2019**, *61*, 245. |
| SCoNC | 1.05 | 0.91 | 1.54 | 0.63 | *Adv. Energy Mater.*  **2019**, *9*, 1900149. |
| Co-N,B-CSs | 0.96 | 0.83 | 1.66 | 0.83 | *ACS Nano*  **2018**, *12*, 1894. |
| Co-POC | 0.94 | 0.83 | 1.70 | 0.87 | *Adv. Mater.*  **2019**, *31*, 1900592. |
| Co-SAs@NC | 0.96 | 0.82 |  |  | *Angew. Chem. Int. Ed.*  **2019**, *58*, 5359. |
| NC-Co SA | 1.0 | 0.87 | 1.59 | 0.72 | *ACS Catal.*  **2018**, *8*, 8961. |
| Fe-N_4_ SAs/NPC | 0.972 | 0.88 | 1.66 | 0.78 | *Angew. Chem. Int. Ed.*  **2018**, *57*, 8614. |
| Fe-N/P-C-700 | 0.94 | 0.87 | 1.66 | 0.79 | *J. Am. Chem. Soc.*  **2020**, *142*, 2404. |
| Fe-N_x_-C | 1.08 | 0.91 | 1.83 | 0.92 | *Adv. Funct. Mater.*  **2019**, *29*, 1808872. |
| NCA_C-Zn_/Fe | 1.10 | 0.89 | 1.60 | 0.71 | *Research*  **2019**, *6813585*, 1. |
| FePc-GO | 0.99 | 0.89 | 1.65 | 0.76 | *ACS Nano*  **2020**, *14*, 13279. |
| MnSAC | 1.04 | 0.91 | 1.58 | 0.67 | *Nano Lett.*  **2020**, *20*, 5443. |
| Ni-N_4_/GHSs/Fe-N_4_ | 0.93 | 0.83 | 1.62 | 0.79 | *Adv. Mater.*  **2020**, *32*, 2003134. |
| CoNi-SAs/NC | 0.88 | 0.76 | 1.57 | 0.81 | *Adv. Mater.*  **2019**, *31*, 1905622. |
| FeCo-N_x_-CN | 0.95 | 0.89 | 1.67 | 0.78 | *Angew. Chem. Int. Ed.*  **2018**, *57*, 1856. |
| G@POF-Co | 0.87 | 0.81 | 1.66 | 0.85 | *Adv. Funct. Mater.*  **2019**, *29*, 1901301. |
| Fe_3_C-Co/NC | 0.94 | 0.88 | 1.60 | 0.72 | *Adv. Funct. Mater.*  **2019**, *29*, 1901949. |
| Co@hNCTs-800 | 0.99 | 0.87 | 1.63 | 0.76 | *Nano Energy*  **2020**, *71*, 104592. |
| CoFe/N-HCSs | 0.90 | 0.79 | 1.52 | 0.73 | *Chem. Eng. J.*  **2021**, *407*, 127961. |
| FeCo/Co_2_P@NPCF | 0.85 | 0.79 | 1.56 | 0.77 | *Adv. Energy Mater.*  **2020**, *10*, 1903854. |
| MnO/Co/PGC | 0.95 | 0.78 | 1.54 | 0.76 | *Adv. Mater.*  **2019**, *31*, 1902339. |
| FeP/Fe_2_O_3_@NPCA | 0.98 | 0.78 | 1.57 | 0.79 | *Adv. Mater.*  **2020**, *32*, 2002292. |
| Pd/FeCo | 0.98 | 0.84 | 1.55 | 0.71 | *Adv. Energy Mater.*  **2020**, *11*, 2002204. |
| Co_2_FeO_4_/NCNTs | 0.94 | 0.80 | 1.65 | 0.85 | *Angew. Chem. Int. Ed.*  **2019**, *58*, 13291. |
| Co/N@CNTs@  CNMF | 0.99 | 0.86 | 1.54 | 0.68 | *Adv. Funct. Mater.*  **2020**, *30*, 2003407. |
| Co-Co_3_O_4_@NAC | 0.94 | 0.80 | 1.61 | 0.81 | *Appl. Catal. B: Environ.*  **2020**, *260*, 118118. |
| f-FeCo-CNT | 0.96 | 0.87 | 1.71 | 0.84 | *Nano Res.*  **2020**, *13*, 1090. |
| CoFe20@CC | 1.02 | 0.86 | 1.52 | 0.66 | *Adv. Mater.*  **2019**, *31*, 1904689. |
| CoNC | 0.97 | 0.87 | 1.55 | 0.68 | *Chem. Eng. J.*  **2021**, *404*, 121112. |
| Co_3_O_4-x_/NG | 0.96 | 0.84 | 1.53 | 0.69 | *Appl. Catal. B: Environ.*  **2020**, *278*, 119300. |
| CoDNG900 | 0.94 | 0.86 | 1.61 | 0.75 | *Appl. Catal. B: Environ.*  **2021**, *281*, 119514. |
| ZOMC | 0.96 | 0.85 | 1.56 | 0.71 | *Adv. Mater.*  **2020**, *32*, 2002170. |
| N-NiSe_2_/CC | 0.80 | 0.73 | 1.5 | 0.77 | *Chem. Eng. J.*  **2020**, *401*, 126088. |
| CoNi/BCF | 0.91 | 0.80 | 1.60 | 0.80 | *Appl. Catal. B: Environ.*  **2019**, *240*, 193. |
| ZIF@HMCS | 0.92 | 0.82 | 1.63 | 0.81 | *Nat. Sci. Rev.*  **2020**, *7*, 609. |
| f-FeCo-CNT | 0.96 | 0.87 | 1.71 | 0.84 | *Nano Res.*  **2020**, *13*, 1090. |
| Co_3_O_4_-Co@NC | 0.97 | 0.86 | 1.58 | 0.72 | *Nano Res.*  **2021**, *14*, 2353. |
| CuCo_2_O_4_@C | 0.95 | 0.85 | 1.56 | 0.71 | *Nano Lett.*  **2017**, *17*, 7989. |
| GNCNTs | 0.92 | 0.85 | 1.60 | 0.75 | *Adv. Funct. Mater.*  **2019**, *30*, 1906081. |
| C-MOF-C2-900 | 0.94 | 0.82 | 1.58 | 0.76 | *Adv. Mater.*  **2018**, *30*, 1705431. |
| Fe_0.5_Ni_0.5_@N-GR | 0.94 | 0.83 | 1.53 | 0.70 | *Adv. Funct. Mater.*  **2018**, *28*, 1706928. |
| CMO/S | 0.92 | 0.76 | 1.7 | 0.94 | *Adv. Energy Mater.*  **2018**, *8*, 1800612. |
| CNF@Zn/CoNC | 0.91 | 0.82 | 1.7 | 0.88 | *Small*  **2018**, *14*, 1704207. |
| CoS_x_/Co-NC-800 | 0.95 | 0.80 | 1.54 | 0.74 | *Adv. Funct. Mater.*  **2019**, *29*, 1904481. |

**Supplementary Table 3.** Lose or gain of electrons for each atom in Co-N_4_ from Bader charge analysis

| Atoms | No adsorption | | OH adsorption | |
| --- | --- | --- | --- | --- |
|  | Planar Co-N_4_ | Curved Co-N_4_ | Planar Co-N_4_ | Curved Co-N_4_ |
| Co | -1.08395 | -1.10007 | -1.43799 | -1.31096 |
| N1 | 2.574933 | 2.675851 | 2.558905 | 2.782011 |
| N2 | 2.574935 | 2.676346 | 2.558941 | 2.78261 |
| N3 | 2.574933 | 2.675851 | 2.558939 | 2.78201 |
| N4 | 2.574935 | 2.676346 | 2.558941 | 2.78261 |

**Supplementary Table 4.** The performance comparison of reported aqueous ZABs.

| Catalysts | OCV  (V) | Peak power density (mW cm^-2^) | Specific capacity (mAh g^-1^) | Cycling stability  (h) | Reference |
| --- | --- | --- | --- | --- | --- |
| Co SA-NDGs | 1.53 | 251.4 | 757.4 | 300 | This work |
| CNT@SAC-Co/NCP | 1.45 | 172 |  | 33 | *Adv. Funct. Mater.*  **2021**, *31*, 2103360. |
| Fe/Ni-N_x_/OC | 1.52 | 148 | 712 | 315 | *Adv. Mater.*  **2020**, *32*, 2004670. |
| PFN | 1.45 | 175 | 816 | 500 | *J. Am. Chem. Soc.*  **2021**, *143*, 11595. |
| Fe-NC SAC |  | 180 | 786 | 53 | *J. Mater. Chem. A*  **2020**, *8*, 9981. |
| Co-N,B-CSs | 1.4 | 100.4 |  | 14 | *ACS Nano*  **2018**, *12*, 1894. |
| Fe_AC_@Fe_SA_-N-C | 1.41 | 115 |  |  | *ACS Nano*  **2019**, *13*, 11853. |
| Cu ISAS/NC |  | 280 | 736 | 50 | *Nat. Commun.*  **2019**, *10*, 3734. |
| MnSAC |  | 258 | 675 |  | *Nano Lett.*  **2020**, *20*, 5443. |
| Co-N_4_/NC | 1.36 | 101.6 | 762.8 | 16.7 | *Nano-Micro Lett.*  **2021**, *13*, 60. |
| Fe/N-G-SAC |  | 120 |  | 240 | *Adv. Mater.*  **2020**, *32*, 2004900. |
| Co SANC-850 | 1.48 |  |  | 44 | *J. Mater. Chem. A*  **2020**, *8*, 2131. |
| Cu-SA/SNC |  | 220 |  |  | *Energy Environ. Sci.*  **2019**, *12*, 3508. |
| Fe SAs/N-C |  | 225 |  | 260 | *ACS Catal.*  **2019**, *9*, 2158. |
| Co_2_FeO_4_/NCNTs | 1.43 | 90.1 |  | 100 | *Angew. Chem. Int. Ed.*  **2019**, *58*, 13291. |
| (Mg,Co)_3_O_4_@  NGC | 1.45 | 125 |  | 200 | *ACS Energy Lett.*  **2017**, *2*, 2706. |
| NCA_C-Zn_/Fe | 1.50 | 231 | 780 | 122 | *Research*  **2019**, *6813585*, 1. |
| Fe-SAs/NP-C | 1.45 | 195 |  |  | *Nat. Commun.*  **2018**, *9*, 5422. |
| S,N-Fe/N/C-CNT | 1.35 | 102.7 |  | 33 | *Angew. Chem. Int. Ed.*  **2017**, *56*, 610. |
| Mo SACs/N-C | 1.47 | 78 | 750 | 120 | *Nano Energy*  **2020**, *67*, 104288. |
| Fe,Mn,N-FGC | 1.41 | 220 |  | 12.8 | *Inorg. Chem.*  **2020**, *59*, 5194. |
| N-CoS_2_ YSSs | 1.41 | 81 | 744 | 165 | *Adv. Sci.*  **2020**, *7*, 2001178. |
| Fe/N-CNRs |  | 181.8 | 771.77 |  | *Adv. Funct. Mater.*  **2020**, *31*, *2008085.* |
| Co-POC |  | 78 |  | 79 | *Adv. Mater.*  **2019**, *31*, 1900592. |
| SilkNC/KB | 1.43 | 91.2 | 614.7 | 33 | *Chem. Mater.*  **2019**, *31*, 1023. |
| Co-SAs@NC | 1.46 | 105.3 |  |  | *Angew. Chem. Int. Ed.* **2019**, *58*, 5359. |
| CoNi/BCF | 1.44 | 155.1 | 710.9 | 30 | *Appl. Catal. B: Environ.*  **2019**, *240*, 193. |
| CoN_4_/NG | 1.51 | 115 | 730 | 100 | *Nano Energy*  **2018**, *50*, 691. |
| CoSA/N,S-HCS | 1.50 | 173.1 | 781 | 333 | *Adv. Energy Mater.*  **2020**, *10*, 2002896. |
| CuSA@HNCN_x_ | 1.51 | 212 | 806 | 300 | *Appl. Catal. B: Environ.*  **2020**, *268*, 118746. |
| N-Co_3_O_4_@NC | 1.344 | 174.1 |  | 50 | *Adv. Funct. Mater.*  **2019**, *29*, 1902875. |
| Cu_1_/NC-900 | 1.48 | 223 |  | 100 | *Nano Res.*  **2021**, *14*, 998. |
| Fe-Zn-SA/NC |  | 167.2 | 756.6 | 120 | *Nano Res.*  **2021**, *14*, 1374. |
| f-FeCo-CNT | 1.484 | 195.8 | 754 | 180 | *Nano Res.*  **2020**, *13*, 1090. |
| Co-Co_3_O_4_@NAC | 1.45 | 164 | 721 | 35 | *Appl. Catal. B: Environ.*  **2020**, *260*, 118118. |
| (Co,Fe)_3_N_R |  | 234 |  | 300 | *Nat. Commun.*  **2020**, *11*, 1952. |
| Fe-N_x_-C | 1.51 | 96.4 | 641 |  | *Adv. Funct. Mater.*  **2019**, *29*, 1808872. |
| Fe-N/P-C-700 | 1.42 | 133.2 | 723.6 | 40 | *J. Am. Chem. Soc.*  **2020**, *142*, 2404. |
| *pf*SAC-Fe | 1.41 | 123.4 | 732 |  | *Sci. Adv.*  **2019**, *5*, eaaw2322. |
| SCoNC | 1.49 | 194 |  | 20 | *Adv. Energy Mater.*  **2019**, *9*, 1900149. |
| A-Co@CMK-3-D |  | 162 | 765 | 45 | *Small Methods*  **2019**, *3*, 1800450. |
| CoFe20@CC | 1.50 | 190.3 | 787.0 | 132 | *Adv. Mater.*  **2019**, *31*, 1904689. |
| Mn-SAS/CN |  | 220 |  |  | *Adv. Energy Mater.* **2021**, *11*, 2002753. |
| AlNiCoRuMo | 1.48 | 146.5 |  | 500 | *ACS Mater. Lett.*  **2020**, *2*, 1698. |
| Co_3_O_4-x_/NG | 1.49 | 166 | 700.6 | 62 | *Appl. Catal. B: Environ.*  **2020**, *278*, 119300. |
| H-Co@FeCo/N/C | 1.45 | 125.2 |  | 200 | *Appl. Catal. B: Environ.*  **2020**, *278*, 119259. |
| Co_3_O_4_-Co@NC |  | 158 | 758 | 200 | *Nano Res.*  **2021**, *14*, 2353. |
| FePc@N,P-DC | 1.45 | 120 | 585 | 50 | *Appl. Catal. B: Environ.*  **2020**, *265*, 118198. |
| NPSC-Co_2_Fe_1_ | 1.44 | 174.6 |  | 70 | *Appl. Catal. B: Environ.*  **2020**, *260*, 118594. |
| CoFe@NC-AS | 1.58 | 102 |  | 48 | *J. Power Sources*  **2020**, *455*, 227975. |
| NPMC/CoFe | 1.49 | 146.3 |  | 75 | *J. Power Sources*  **2019**, *441*, 227177. |
| ZIF/HMCS | 1.38 | 120.2 |  | 80 | *Nat. Sci. Rev.*  **2020**, *7*, 609. |
| CoFe/N-HCSs | 1.39 | 96.5 | 774.5 |  | *Chem. Eng. J.*  **2021**, *407*, 127961. |
| PdMo bimetallene | 1.48 | 154.2 | 798 | 500 | *Nature*  **2019**, *574*, 81. |
| SA-PtCoF |  | 125 | 808 | 240 | *Energy Environ. Sci.*  **2020**, *13*, 884. |
| FeP/Fe_2_O_3_ @NPCA |  | 130 | 717 | 200 | *Adv. Mater.*  **2020**, *32*, 2002292. |
| ZOMC | 1.49 | 221.1 | 697.9 | 165 | *Adv. Mater.*  **2020**, *32*, 2002170. |
| Co/N@CNTs@  CNMF | 1.52 | 133 |  | 190 | *Adv. Funct. Mater.*  **2020**, *30*, 2003407. |
| CoO_x_/NMC | 1.48 | 195.3 |  | 400 | *Energy Storage Mater.*  **2020**, *29*, 156. |
| CoFe@N-GCNCs | 1.45 | 132.5 |  | 100 | *J. Power Sources*  **2020**, *480*, 229107. |
| FeCo/Co_2_P@  NPCF | 1.44 | 154 |  | 107 | *Adv. Energy Mater.*  **2020**, *10*, 1903854. |
| Ni\|MnO/CNF | 1.56 | 138.6 |  | 120 | *Adv. Funct. Mater.*  **2020**, *30*, 1910568. |
| CoFe/N-GCT | 1.43 | 203 |  | 267 | *Angew. Chem. Int. Ed.*  **2018**, *57*, 16166. |
| Mo-N/C@MoS_2_ | 1.46 | 196.4 |  | 48 | *Adv. Funct. Mater.*  **2017**, *27*, 1702300. |
| f-FeCo-CNT | 1.484 | 195.8 | 754 | 180 | *Nano Res.*  **2020**, *13*, 1090. |
| Co-NC-800 | 1.44 | 109.5 | 657.2 | 36 | *Chem. Eng. J.*  **2021**, *404*, 121112. |
| CoDNG900 | 1.45 | 207 | 669 | 666 | *Appl. Catal. B: Environ.*  **2021**, *281*, 119514. |
| FeCo@MNC | 1.41 | 115 |  | 24 | *Appl. Catal. B: Environ.*  **2019**, *244*, 150. |
| Ni_0.2_Co_0.8_Se | 1.44 | 223.5 | 698.6 | 50 | *Nano-Micro Lett.*  **2019**, *11*, 28. |
| Meso-CoNC@GF | 1.51 | 154.4 |  | 105 | *Adv. Mater.*  **2017**, *30*, 1704898. |
| Fe_0.5_Ni_0.5_@N-GR | 1.48 | 85 | 765 | 40 | *Adv. Funct. Mater.*  **2018**, *28*, 1706928. |
| C-MOF-C2-900 | 1.46 | 105 | 741 | 120 | *Adv. Mater.*  **2018**, *30*, 1705431. |
| CNF@Zn/CoNC | 1.46 | 140.1 | 680.2 | 150 | *Small*  **2018**, *14*, 1704207. |
| CoS_x_/Co-NC-800 | 1.40 | 103 | 770.4 | 200 | *Adv. Funct. Mater.*  **2019**, *29*, 1904481. |
| N, S-CC | 1.36 | 42 |  |  | *Adv. Sci.*  **2018**, *5*, 1800760. |
| o-CC-H_2_ |  | 91.4 | 707 | 30 | *Energy Storage Mater.*  **2018**, *15*, 124. |
| CNT@POF |  | 237 | 772.7 | 67 | *Energy Environ. Sci.*  **2018**, *11*, 1723. |
| N-GQDs/  NiCo_2_S_4_/CC |  | 75.2 |  | 200 | *Small*  **2019**, *15*, 1903610. |
| NGM-Co |  | 152 | 750 | 60 | *Adv. Mater.*  **2017**, *29*, 1703185. |
| Co-N-CNT | 1.37 | 101 |  | 15 | *Adv. Funct. Mater.*  **2017**, *28*, 1705048. |

**Supplementary Table 5.** Performance comparison of reported quasi-solid-state ZABs.

| Catalysts | Gel electrolyte | OCV  (V) | Peak power density (mW  cm^-2^) | Cycling time (h)  @current density (mA cm^-2^) | Reference |
| --- | --- | --- | --- | --- | --- |
| Co SA-NDGs | PAM | 1.43 | 219.9 | 50@100  100@50 | This work |
| Co SA@NCF/CNF | PVA | 1.41 |  | 9@3 | *Adv. Mater.*  **2019**, *31*, 1808267. |
| NiCo_2.148_O_4_ PNS | PVA | 1.30 |  | 20@1 | *Adv. Mater.*  **2020**, *32*, 2001651. |
| Ce-LaCoO_3_ | PVA | 1.33 | 31 | 8@2 | *Nano Energy*  **2020**, *50*, 691. |
| CoN_4_/NG | PVA |  | 28 | 6@3 | *Nano Energy*  **2018**, *50*, 691. |
| CoSA/N,S-HCS | PVA | 1.48 |  | 17@5 | *Adv. Energy Mater.*  **2020**, *10*, 2002896. |
| Fe-NC SAC | PVA | 1.424 | 45 | 1@2 | *J. Mater. Chem. A*  **2020**, *8*, 9981. |
| Mn-CoN | PAAm | 1.37 | 48 | 20@2 | *Sci. China Chem.*  **2020**, *63*, 7. |
| Mn_3_O_4_/NiCo_2_S_4_ | PVA-PEO | 1.427 |  | 16.8@1 | *J. Power Sources*  **2020**, *462*, 228162. |
| NiCo_2_O_4_/MXene | PVA | 1.40 | 55.1 | 33@1 | *ACS Appl. Mater. Interfaces* **2020**, *12*, 44639. |
| NPF@CNF-800 | PVA | 1.33 | 64 | 20@5 | *ACS Appl. Mater. Interfaces*  **2021**, *13*, 13328. |
| CoS/CoO@NGNs | PVA | 1.3 | 39.3 | 10@1 | *Nano-Micro Lett.*  **2021**, *13*, 3. |
| NCNTM | PAAs | 1.49 | 176 | 30@1 | *J. Energy Chem.*  **2021**, *55*, 183. |
| FeCo/Se-CNT | PVA | 1.41 | 37.5 | 20@5 | *Nano Lett.*,  **2021**, *21*, 2255. |
| CoFe/N-HCSs | PVA | 1.4 |  | 10@2 | *Chem. Eng. J.*  **2021**, *407*, 127961. |
| N-Mo-holey G | PANa | 1.37 | 83 | 88@2 | *Appl. Catal.B: Environ.*  **2020**, *276*, 119172. |
| AlFeCoNiCr | PANa | 1.38 | 100 | 60@2 | *Appl. Catal. B: Environ.* **2020**, *268*, 118431. |
| FePc@N,P-DC | PVA | 1.33 |  | 8@2 | *Appl. Catal. B: Environ.*  **2020**, *260*, 118198. |
| S-Ni_3_FeN/NSG | PAAs | 1.38 | 140.1 | 35@1 | *Appl. Catal. B: Environ.*  **2020**, *274*, 119086. |
| Fe_0.5_Ni_0.5_@N-GR | PVA | 1.352 |  | 18@10 | *Adv. Funct. Mater.*  **2018**, *28*, 1706928. |
| N-NiSe_2_/CC | PVA |  | 27 | 30@1 | *Chem. Eng. J.*  **2020**, *401*, 126088. |
| FeP/Fe_2_O_3_@  NPCA | PVA | 1.42 | 40.8 | 8@5 | *Adv. Mater.*  **2020**, *32*, 2002292. |
| FeCo/Co_2_P@  NPCF | PVA | 1.26 |  | 15@2 | *Adv. Energy Mater.*  **2020**, *10*, 1903854. |
| Co_3_O_4_@*_x_*HoNPs@  HPNCS | PVA | 1.459 | 94.1 | 18@3 | *Angew. Chem. Int. Ed.*  **2019**, *58*, 13840. |
| GNCNTs | PAA | 1.54 | 223 | 24@1 | *Adv. Funct. Mater.*  **2019**, *30*, 1906081. |
| Co-NC@Al_2_O_3_ | PAM | 1.41 | 72.4 | 10@20 | *Adv. Mater.*  **2018**, *30*, 1805268. |
| N-GQDs/  NiCo_2_S_4_/CC | PVA | 1.406 | 26.2 | 12@20 | *Small*  **2019**, *15*, 1903610. |
| Meso-CoNC@GF | PVA | 1.40 | 85.6 | 12@20 | *Adv. Mater.*  **2017**, *30*, 1704898. |
| NC-Co_3_O_4_-90 | PAM | 1.44 | 82 | 21@5 | *Adv. Mater.*  **2017**, *29*, 1704117. |
| N, S-CC | PVA-PEO | 1.247 | 47 | 8@5 | *Adv. Sci.*  **2018**, *5*, 1800760. |
| CNT@POF | PVA | 1.39 | 22.3 | 4@1 | *Energy Environ. Sci.*  **2018**, *11*, 1723. |
| Co_3_O_4_/CC | PVA-SiO_2_ | 1.27 | 62.6 | 48@3 | *Nano Energy*  **2019**, *56*, 454. |
| CMO/S | PVA-PEO | 1.32 |  | 10@1 | *Adv. Energy Mater.*  **2018**, *8*, 1800612. |
| NGM-Co | PVA | 1.439 | 28 | 1@1 | *Adv. Mater.*  **2017**, *29*, 1703185. |
| NP-Co_3_O_4_/CC | PVA | 1.349 | 99.8 | 20@5 | *Energy Storage Mater.*  **2020**, *26*, 157. |
| Ni_0.2_Co_0.8_Se | PVA | 1.428 | 41 | 8@2 | *Nano-Micro Lett.*  **2019**, *11*, 28. |
| Co_3_O_4_/N-CNT | PVA | 1.31 |  | 20@2 | *Small*  **2017**, *13*, 1700518. |
| SilkNC/KB | PVA |  | 32.3 | 10@1 | *Chem. Mater.*  **2019**, *31*, 1023. |
| Co/ZnCo_2_O_4_@  NC-CNTs | PVA | 1.30 | 151 | 21@5 | *Nano Energy*  **2021**, *82*, 105710. |
| D-CMO | PANa | 1.46 | 149 | 34@2 | *Nano Energy*  **2021**, *85*, 106020. |
| Co_3_O_4_/Mn_3_O_4_/  CN_x_@CNFs | PVA | 1.51 | 191 | 9@1 | *Electrochim. Acta*  **2020**, *344*, 136145. |
| HCA-Co | PAA | 1.40 | 44.8 | 40@1 | *Chem. Eng. J.*  **2019**, *369*, 988. |
| CoNC | PAAS | 1.33 | 117 | 21@2 | *Chem. Eng. J.*  **2021**, *404*, 127112. |
| Fe-N-C-700 | PAA | 1.42 | 70 |  | *Chem. Eng. J.*  **2021**, *405*, 125956. |
| NiFe@N-CFs | PVA | 1.18 |  | 10@1 | *J. Mater. Chem. A*  **2020**, *8*, 13725. |
| Co-Fe-S@NSRPC | PAM-PAA | 1.42 | 78 | 50@5 | *Nanoscale*  **2020**, *12*, 11746. |
| Mn_3_O_4_/NiCo_2_S_4_ | PVA/PEO | 1.43 |  | 16.8@1 | [*J. Power Sources*](https://www.sciencedirect.com/science/journal/03787753)  [**2020**, *462*](https://www.sciencedirect.com/science/journal/03787753/462/supp/C), 228162. |
| Co/Co-N-C | PVA | 1.41 |  | 10@2 | *Adv. Mater.*  **2019**, *31*, 1901666. |
| Co-NCNT | PANa | 1.45 | 144.6 | 75@2 | [*Energy Storage Mater.*](https://www.sciencedirect.com/science/journal/24058297) [**2019**, *20*](https://www.sciencedirect.com/science/journal/24058297/20/supp/C), 234. |
| Co-N_x_-YSC-600 | PVA | 1.35 | 55.3 | 4@5 | *Nano Energy* 2021, *89*, 106314. |
| WN-Ni@  N,P-CNT | PVA | 1.57 | 100.4 | 10@1 | *Appl. Catal. B: Environ.*2021, *298*, 120511. |
| Fe_1_Co_1_-CNF | PVA |  |  | 3@2 | *Nano Energy* 2021, *87*, 106147. |
| Co/CoO@NSC | PANa | 1.43 | 82.7 | 143@2 | *J. Energy Chem.*2022, *64*, 385. |
| NiFe/N-CNT | PVA | 1.41 | 105.4 | 10@1 | *Nano Energy* 2020, *68*, 104293. |
| OCNT | PAA | 1.39 | 103 | 17@5 | *Energy Storage Mater.* 2020, *30*, 138. |
| FeCo/N-CNTs@CC | PVA | 1.40 | 127 | 8@20 | *ACS Sustainable Chem. Eng.* 2021, *9*, 4498. |
| FeS_2_-CoS_2_/NCFs | PVA | 1.39 | 69 | 20@1 | *J. Power Sources* 2021, *482*, 228955. |
| CoNC-MOG | PVA | 1.41 | 63 | 12@2 | *Appl. Surf. Sci.*2021, *537*, 147818. |
| NBSCF | CNF-based membranes | 1.44 |  | 15@1 | *J. Mater. Chem. A* 2019, *7*, 24231. |
| CoFe@NCNT/  CFC | PVA | 1.43 | 37.7 | 15@1 | *J. Mater. Chem. A*2020, *8*, 18162. |
| Ni_5.7_Ru_0.3_ | PVA | 1.33 | 98.3 | 68@2 | *Chem. Commun.*2020, *56*, 13615. |
| NC-Co/CoNx | PAA | 1.40 | 41.5 | 25@1 | *Energy Storage Mater.*2019, *16*, 243. |
| NO-G@CP | PVA | 1.33 | 65.1 | 6@5 | *J. Mater. Chem. A*2020, *8*, 11202. |
| CoO-NSC | PAA |  | 65 | 35@1 | *ACS Appl. Mater. Interfaces* 2019, *11*, 16720. |
| P-O/FeN_4_-CNS | PVA | 1.41 | 109 | 20@10 | *ACS Appl. Mater. Interfaces* 2019, *11*, 33054. |
| CoFe/FeNC | PAA | 1.47 | 108.6 | 18@2 | *ACS Sustainable Chem. Eng.* 2020, *8*, 9009. |
| FeCo-N-C-700 | PVA | 1.43 |  | 62@10 | *J. Mater. Chem. A*2020, *8*, 9355. |
| FeN_x_/N,S-C | PANa |  | 70.6 | 52@5 | *Carbon*2020, *166*, 64. |
| Fe-NC SAC | PVA | 1.42 | 45 | 1@1 | *J. Mater. Chem. A*2020, *8*, 9981. |
| N-CuCoS_1.97_ NWs | PVA | 1.36 |  | 8@1 | *J. Energy Chem.*2019, *34*, 1. |
| CoFeP@C | PVA |  | 72.6 | 20@1 | *ACS Appl. Mater. Interfaces* 2021, *13*, 22282. |
| CoIn_2_Se_4_ | PVA | 1.37 | 107 | 68@10 | *ACS Appl. Mater. Interfaces* 2020, *12*, 8115. |
| V-Co_3_O_4_ | PVA | 1.39 | 40.6 | 32@7 | *ACS Catal.* 2021, *11*, 8097. |
| N-NiCo_2_O_4_ | PVA |  | 23 | 53@5 | *ACS Appl. Energy Mater.* 2019, *2*, 2296. |
| h-FeCo alloy/NCNFs | PAA | 1.34 | 12.6 | 19@1 | *Sustainable Energy Fuels* 2020, *4*, 1747. |
| BFC-FC-0.2 | PAA | 1.49 | 160 | 110@2 | *Angew. Chem. Int. Ed.*2020, *59*, 4793. |
| FeNi SAs/NC | PAA | 1.45 | 42.2 | 9@3 | *Adv. Energy Mater.*2021, *11*, 2101242. |
| Fe/Fe_3_C@  NdC-NCs | P-(AM-co-AA) | 1.43 | 60 | 40@5 | *J. Mater. Chem. A*2019, *7*, 17581. |
| FeCu-N-HC | PVA | 1.41 | 113 | 110@10 | *Adv. Funct. Mater.*2020, *31*, 2006533. |
| Fe-N-C | PANa-cellulose | 1.48 | 108.6 | 110@5 | *Adv. Energy Mater.*2019, *9*, 1803046. |
| CoNCNTF/CNFs | PVA | 1.30 | 63 | 11@0.5 | *Carbon*2019, *142*, 379e387. |
| NiS_2_/CoS_2_ | PVA |  | 101 | 33@10 | *J. Power Sources*2019, *437*, 226893. |
| Pt/RuO_2_/CF | PAM-CNF/KOH/KI | 1.45 | 65 | 75@2 | [*Energy Storage Mater.*](https://www.sciencedirect.com/science/journal/24058297) [**2021**, *42*](https://www.sciencedirect.com/science/journal/24058297/42/supp/C), 88. |
| Co_3_O_4_/CC | KI-PVAA-GO GPE |  | 78.6 | 200@2 | *Adv. Mater.*2020, *32*, 1908127. |
| Ni-Co_9_S_8_/rGN | PAA | 1.37 | 110 | 25@1 | *Appl. Catal. B: Environ.*2021, *298*, 120539. |
| NiCo_2_O_4_@  N-CNWs | PVA-PEO | 1.28 |  | 45@1 | *Electrochim. Acta*2019, *319*, 1e9. |
| CoSe_2_-NCNT NSA | PAA | 1.37 | 51.1 | 5@2 | *Nanoscale*2021, *13*, 3019. |
| P-CoSe_2_/N-C FAs | PAA | 1.30 |  | 20@1 | *Adv. Funct. Mater.*2018, *28*, 1804846. |
| PdNi/Ni@N-C | PAA | 1.40 | 66.5 | 15@1 | *Energy Storage Mater.*2021, *42*, 118. |
| ODAC-CoO-30 | PVA | 1.41 | 42 | 12@2 | *Adv. Funct. Mater.*2021, *31*, 2101239. |
| Co-NDC | PVA |  | 45.9 | 3.3@2 | *Sci. Bull.*2018, *63*, 548. |

**Supplementary Table 6.** Performance comparison of low-temperature electrochemical device.

| Energy devices | Electrolyte | Working tempera-ture (^o^C) | Battery  performance | Reference |
| --- | --- | --- | --- | --- |
| Zinc-air battery | PAM organohydrogel electrolyte | -60 | 300 h@0.5 mA cm^-2^  100 h@1.0 mA cm^-2^ | This work |
| Zinc-air battery | PAM organohydrogel electrolyte | -40 | 21.9 mW cm^-2^; Specific capacity of 778.4 mAh g^-1^; Energy density of 918.5 Wh kg^-1^;  160 h@1.0 mA cm^-2^ | This work |
| Zinc-air battery | CsOH-based electrolyte | -10 | 57.9 mW cm^-2^;  160 cycles@5.0 mA cm^-2^; 65 cycles@10.0 mA cm^-2^ at -10 °C;  6.5 mW cm^-2^ at -40 °C | *Angew. Chem. Int. Ed.* **2021**, *60*, 15281. |
| Zinc-air battery | PAA hydrogel electrolyte | -20 | 80.5 mW cm^-2^; Specific capacity of 691 mAh g^-1^; Energy density of 798 Wh kg^-1^ | *Angew. Chem. Int. Ed.*  **2020**, *59*, 4793. |
| Zinc-air battery | CBCs super-ion conductors | -20 | 2 h of discharge at -20 °C | *Nat. Energy*  **2021**, *6*, 592. |
| Zinc-air battery | PAM/PAA  organohydrogel electrolyte | -20 | 1.44 V of OCV for 0.5 h at -20 °C;  10 h@1 mA cm^-2^ | *ACS Sustainable Chem. Eng.* **2020**, *8*, 11501. |
| Zinc-air battery | PAMC | -20 | 35.8 mW cm^-2^;  190 cycles@2 mA cm^-2^ at -30 °C | *Chem. Eng. J.*  **2021**, *417*, 129179. |
| Zinc-air battery | A-PAA hydrogel electrolyte | -30 | 63.6 mW cm^-2^; Specific capacity of 699 mAh g^-1^; Energy density of 789 Wh kg^-1^; 500 cycles@2 mA cm^-2^ at -30 °C | *Energy Environ. Sci.* **2021**, *14*, 4926. |
| Zinc-air battery | PVA organohydrogel electrolyte | -35 | 1.25 V of OCV for 120 h at -35 °C; 8.2 mW cm^-2^; | *Adv. Mater.*  **2020**, *32*, 2001651. |
| Zinc-air battery | PAM-CNF/KOH/KI-based hydrogel | -40 | 10 mW cm^-2^  Specific capacity of 743 mAh g^-1^;  45 h@2 mA cm^-2^ | [*Energy Storage Mater.*](https://www.sciencedirect.com/science/journal/24058297) [**2021**, *42*](https://www.sciencedirect.com/science/journal/24058297/42/supp/C), 88. |
| Zinc-air battery | SP-DN hydrogel electrolyte | -50 | 1.38 V of OCV; 97 mW cm^-2^; Specific capacity of 620 mAh g^-1^ | *Energy Environ. Sci.*2021, *14*, 4451. |
| Zn/LiFePO_4_ hybrid battery | ZL-PAAm hydrogel electrolyte | -20 | 98% capacity retention upon cooling down to -20 °C; near 100% capacity retention with >99.5%; Coulombic efficiency over 500 cycles at -20 °C | *Adv. Funct. Mater.*  **2019**, *30*, 1907218. |
| Strain sensors | MXene nanocomposite organohydrogel | -40 | A broad strain range (up to 350% strain) and a high gauge factor of 44.85 at -40 °C | *Adv. Funct. Mater.*  **2019**, *29*, 1904507. |
| Super-capacitors | PVA organohydrogel | -40 | 70.6% capacitance retained at -40 °C and 11.7% capacitance decay over 5000 charge/discharge cycles at -20 °C | *Adv. Energy Mater.*  **2018**, *8*, 1801967. |
| Li metal battery | 1 M LiFSI DEE | -60 | Discharge capacity of  236 and 13 mAh g^-1^ at -40 and -60 °C | *Nat. Energy*  **2021**, *6*, 303. |
| Li metal battery | 5 M LiTFSI/EA + DCM | -70 | High energy (178 Wh kg^-1^ and power (2877 W kg^-1^) at -70 ^o^C | *Angew. Chem. Int. Ed.*  **2019**, *58*, 5623. |
| Li metal battery | 1 M LiPF6 MTFP/FEC (9:1) | -60 | Specific capacities of 161, 149, and 133 mAh g^-1^ at -40, -50, and -60 °C | *ACS Energy Lett.*  **2020**, *5*, 1438. |
| Na metal battery | EC/PC-based electrolyte | -30 | Specific capacities of 92.1 mAh g^-1^ at -30 °C | *Energy Environ. Sci.*  **2021**, *14*, 4936. |
| Li-ion battery | EA-based electrolyte | -70 | Specific capacity of 20 mAh g^-1^ at low rate of 0.2 C; 70% of capacity at room temperature | *Joule*, **2018**, *2*, 902. |
| Na-ion battery | PHP5A electrolyte | -20 | Specific capacity of 44.2 mAh g^-1^ | *Adv. Funct. Mater.* **2019**, *30*, 1906770. |
| Zn-ion battery | PAM polyelectrolyte | -20 | Specific capacity of 160.3 mAh g^-1^ and stable 600 cycles at 0.2 A g^-1^ | *Energy Storage Mater.*  **2022**, *44*, 517. |
| Zn-ion battery | 3 M Zn(CF_3_SO_3_)_2_ | -20 | Specific capacity of 120, 96, 64, and 41 mAh g^-1^ at 0.1, 0.2, 0.5, and 1.0 A g^-1^ | *J. Power Sources*  **2019**, *441*, 227192. |
| Zn ion hybrid capacitor | Zn(ClO_4_)_2_ salty ice | -50 | 74.1% of the room temperature capacity at -60 °C; 280 days at 1 A g^-1^ at -30 °C | *Adv. Funct. Mater.*  **2021**, *31*, 2101277. |
| PANI\|LTE\|Zn battery | ZnCl_2_-based electrolyte | -70 | Specific capacity of 84.9 mAh g^-1^ and stable during over 2000 cycles with ~100% capacity retention | *Nat. Commun.*  **2020**, *11*, 4463. |
| Li-CO_2_ battery | DOL-based electrolyte | -60 | Discharge capacity of 8976 mAh g^-1^ and long lifespan of 150 cycles (1500 h) with a fixed 500 mAh g^-1^ capacity per cycle at -60 °C | *Adv. Funct. Mater.*  **2020**, *30*, 2001619. |
| Proton battery | 62 wt% (9.5 M) H_3_PO_4_ | -78 | Stable cycle life for  450 cycles, high round-trip efficiency of 85% | *Adv. Energy Mater.*  **2020**, *10*, 2000968. |
| Proton battery | 2 M H_2_SO_4_  + 2 M MnSO_4_ | -70 | Discharge capacity of  171.8 mAh g^-1^ and 100 cycles at 0.1 A g^-1^ | *ACS Energy Lett.*  **2020**, *5*, 685. |
| Zn battery | 2M Zn(CF_3_SO_3_)_2_ | -30 | Specific capacity of 285.0 mAh g^-1^ at -30 °C and capacity retention of 81.7% after 1000 cycles | *ACS Energy Lett.*  **2021**, *6*, 2704. |
| Li-S battery | AMDS-modified electrolyte | -40 | Specific capacity of 2408 mAh g^-1^ at -30 °C and stable 50 cycles | *ACS Nano*  **2021**, *15*, 13847. |
| Zn-MnO_2_ battery | EG-waPUA/PAM hydrogel | -20 | Specific capacity of 226 mAh g^-1^ at -20 °C and capacity retention of 87.41% over 600 cycles | *Energy Environ. Sci.*  **2019**, *12*, 706. |

**Reference**

[1] Xu, Y. et al. Nickel nanoparticles encapsulated in few-layer nitrogen doped graphene derived from metal-organic frameworks as efficient bifunctional electrocatalysts for overall water splitting. *Adv. Mater.* **29**, 1605957 (2017).

[2] Han, H. et al. Advantageous crystalline-amorphous phase boundary for enhanced electrochemical water oxidation. *Energy Environ. Sci.* **12**, 2443-2454 (2019).

[3] Li, H. et al. Waterproof and tailorable elastic rechargeable yarn zinc ion batteries by a cross-linked polyacrylamide electrolyte. *ACS Nano* **12**, 3140-3148 (2018).

[4] Yang, Q. et al. Dendrites in Zn-based batteries. *Adv. Mater.* **32**, 2001854 (2020).

[5] Zhang, Y. et al. Reaction modifier system enable double-network hydrogel electrolyte for flexible zinc-air batteries with tolerance to extreme cold conditions. *Energy Storage Mater.* **42**, 88-96 (2021).

[6] Dong, X. et al. High-energy rechargeable metallic lithium battery at -70 ^o^C enabled by a cosolvent electrolyte. *Angew. Chem. Int. Ed.* **58**, 5623-5627 (2019).
