# Supplementary material for: Quasi-solid-state Zn-air batteries with an atomically dispersed cobalt electrocatalyst and organohydrogel electrolyte
Source: Nat Commun. 2022 Jun 27;13:3689. doi: 10.1038/s41467-022-31383-4 (PMC9237111; doi:10.1038/s41467-022-31383-4)
Supplement: Supplementary file 3 — Description of Additional Supplementary Information [file 41467_2022_31383_MOESM3_ESM.pdf]

Description of Additional Supplementary Information

Title: Supplementary Movie 1

Description: To understand the evolution process of atomically dispersed Co atoms during 9 pyrolysis, molecular dynamics (MD) simulations was performed. We placed a Co cluster 10 consisting of two Co atoms on a tetrapyridine N-doped carbon site to study the possible changes at 800 o 11 C. The results show that two Co single atoms do not form a stable Co 12 cluster. The Co-Co bond of the cluster gradually becomes longer until it is broken. The 13 Co connected to the tetrapyridine N automatically forms a typical planar Co-N4 14 configuration, while the other Co atom will move further and further away from the Co15 N4 coordination. This result fully proves that stable Co dimers are not easy to form, but 16 due to the synergistic effect of charge transfer and vacancy defects, the typical planar 17 four-coordinate configuration of Co-N4 is formed. Simultaneously, the escaped Co atom 18 may be captured by the doped N species or graphene defects until it is stabilized.
