# Supplementary figures and images for: Quasi-solid-state Zn-air batteries with an atomically dispersed cobalt electrocatalyst and organohydrogel electrolyte
Source: Nat Commun. 2022 Jun 27;13:3689. doi: 10.1038/s41467-022-31383-4 (PMC9237111; doi:10.1038/s41467-022-31383-4)

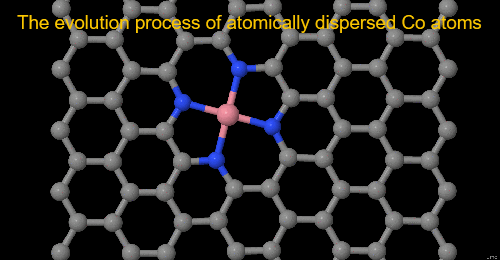

Supplement: Supplementary file 4 — Supplementary Movie 1 [file 41467_2022_31383_MOESM4_ESM.gif]
